# The impact of increasing urban surface albedo on outdoor air and surface temperatures during summer in newly developed areas

**DOI:** 10.1038/s41598-025-08574-2

**Published:** 2025-07-11

**Authors:** Donia Elgendy, Osama Tolba, Tarek Kamel

**Affiliations:** Arab Academy for Science, Technology and Maritime Transport - Heliopolis, Cairo, Egypt

**Keywords:** Surface albedo, Urban geometry, Reflective materials, Urban Heat Island, Urban morphology, Ladybug plugin, Environmental impact, Climate-change mitigation

## Abstract

This study investigates the influence of increasing road surface albedo on outdoor air and surface temperatures in residential areas, taking into account constraints on broader environmental modifications. Urban albedo, which is determined by spatial geometry and material reflectance, influences the amount of solar radiation bouncing back into the atmosphere. Field measurements were conducted on-site to document Air Temperature (T_a_), Wind Speed (WS), Relative Humidity (RH), Mean Radiant Temperature (MRT) providing the basis for validating simulation models. The urban geometry was reconstructed from real site data and simulated using a hybrid modeling approach, combining Ladybug with Grasshopper for Surface Temperature (T_s_), MRT, and Universal Thermal Climate Index simulations, and ENVI-met for T_a_, RH, and WS simulations. ENVI-met outputs were integrated into Grasshopper to achieve high-accuracy environmental modeling. Results demonstrate that increasing pavement albedo from 0.12 to 0.50 reduced T_s_ by up to 12.94 °C at peak solar hours and lowered T_a_ by a maximum of 1.96 °C during the day. The research addresses a critical gap by focusing solely on altering material reflectivity without changing urban morphology or adding any canopies either structured or vegetation. The findings confirm that enhancing surface albedo is an effective method to reduce daytime heat trapping & accumulation, and shortwave radiation absorption which mitigate the Urban Heat Island phenomenon.

## Introduction

**Fig. 1:**
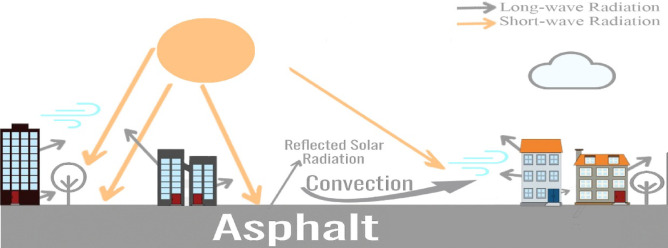
Pavement’s thermal interactions with the surrounding environment.

Cities across the world have grown throughout time, driven by urbanization. The expansion of cities and the rise of dark-coloured surfaces, which absorb shortwave solar radiation during the day and emit long wave heat at night through building envelopes and paved surfaces. Therefore, the Urban Heat Island (UHI) is more pronounced in urban cities areas rather than rural due to the increased air convection with higher Surface Temperatures (T_s_) of asphalt surfaces, leading to increase the Air Temperature (T_a_)^1^. This interaction between pavement materials, surface radiation, and surrounding T_a_ is illustrated in Fig. 1, which highlights the thermal exchanges occurring between urban surfaces and the atmosphere.

UHI is the phenomenon whereby urban regions have notably higher temperatures than their surrounding rural areas^2^. Human health is directly threatened by the high temperatures linked to UHI, which can cause emotional discomfort, heatstroke, and even death^3^. Furthermore, by worsening air pollution and lowering water quality, UHI can have an indirect effect on people’s health^4^. Several thermal mitigation techniques have been put forth in recent research to lessen the impacts of UHI and enhance human thermal comfort^5^.

Roofs and pavements are two examples of urban surfaces that have a notable impact on the urban microclimate, changing its energy balance and causing what is known as the UHI effect. UHIs have been linked to detrimental health effects as well as an increase in energy consumption from buildings. The extent of these consequences is only anticipated to increase with rising global average temperatures^3^. Public and commercial parties have started putting mitigation plans into place to allay these worries. Every UHI mitigation method should ideally avoid reducing urban warming by causing additional environmental impacts; if it does, decision-makers should be able to quantify the decisions that they need to make^6^.

Urban climates vary significantly across different geographic zones, ranging from hot-humid and hot-arid to temperate and cold climates. Each climate zone presents unique challenges in mitigating urban heat, and as a result, the effectiveness of passive strategies such as increasing surface albedo may vary accordingly. In hot-humid zones, reflective materials may need to be paired with ventilation to counteract high moisture levels, while in temperate and cold regions, albedo changes can influence energy demand for heating during winter. Therefore, understanding the role of reflective surfaces across different climatic contexts is essential for tailoring urban heat mitigation strategies that are both environmentally and functionally appropriate^7–9^.

This study specifically focuses on hot-arid climates, where high solar radiation, minimal vegetation, and limited evaporative cooling create critical outdoor thermal challenges. Cairo, Egypt, was selected as a representative case due to its dense urban expansion and newly implemented urban regulations that significantly altered surface composition. In such climates, increasing urban surface albedo presents a promising solution to reduce T_s_ and T_a_ without relying on resource-intensive interventions such as vegetation or water-based cooling. By focusing solely on material reflectivity as the primary intervention, this study provides context-specific insight that are directly relevant to hot-arid contexts and potentially adaptable to other regions facing similar environmental constraints as demonstrated by Abdelwahab et al.^10^.

The overall environmental gain of the most often mentioned mitigation techniques, such as “cool” roofs and more green space, has been extensively discussed. “Cool” pavements are one mitigation technique that hasn’t been thoroughly investigated. Cool pavements change the surrounding environment’s energy balance, usually by having surface properties that reflect more solar radiation. Albedo is the name given to this property, and higher values indicate greater reflectivity. Numerous real-world projects across cities such as Athens, Tirana, and Los Angeles have implemented reflective pavements with measurable success in reducing T_a_. These applications and their outcomes are detailed below in Table 1. In general, pavement albedo is impacted by climate in two different ways. First, energy from the surface is reflected into the surrounding air, creating a direct Radiative Forcing (RF). The second impact is the shift in the energy consumption of adjacent buildings, which is now known as Building Energy Demand (BED), and the corresponding Greenhouse Gas (GHG) releases from energy production^11^.

**Table 1:** Effect of increasing pavement’s albedo on air temperature.

References	Type of existing pavement/Initial Albedo	Type of new pavement/Final Albedo	Results
^26^	Concrete and asphalt pavements that are black and have an albedo below 0.4	Roads with cool asphalt and an albedo of 0.35. Pavements made of natural reflecting materials (marble) have an albedo of 0.7, while concrete pavements painted with cool, infrared reflective paints have an albedo of 0.78	Pavement replacement lowers the average peak T_a_ by 1.2 to 2.0 °C
^27^	Dark concrete and black asphalt pavements with an albedo of less than 0.2	The albedo of concrete pavements painted with infrared reflective cool paints ranges from 0.65 to 0.75, depending on the colour	Pavement replacement lowers the average peak T_a_ by 2.1 °C
^44^	Tiles made from white-coloured concrete with an albedo of 0.45. Roads with black asphalt	Application of photo catalytic asphalt on roadways. Infrared reflective cool paints with an albedo of 0.68 are used for colouring concrete pavements	Pavement replacement lowers the average peak T_a_ by 1.6 °C
^45^	Dark paving materials and asphalt concrete. The paved surfaces had an albedo of 0.35 to 0.45, whereas the portions covered with asphalt and concrete had an albedo of less than 0.2	Infrared reflective cool paints with an albedo of 0.60 are used to colour concrete pavements	A typical summer day’s peak T_a _is lowered by up to 1.9 °C by using cool paving materials
^46^	NA	Paving materials that have an albedo of 0.8	Due to the usage of cool pavement and trees, the T_a_ has decreased by 1.5 °C . It appears that cool pavements contribute about 0.1 °C
^47^	NA	Cool pavements albedo increased by 0.02	Decrease of 0.2 °C in the average T_a_
^47^	NA	Cool pavements albedo increased by 0.09	Decrease of 0.8 °C in the average T_a_
^48^	NA	Cool pavements albedo increased	Decrease of 0.15 °C in the average T_a_
^49^	0.15	0.45	Decrease of 2.5 °C in the average T_a_
^50^	0.117 to 0.152	0.18–0.252	Decrease of 1.0 °C in the average T_a_
^50^	0.117 to 0.152	0.199–0.374	Decrease of 2.0 °C in the average T_a_
^51^	0.13	0.26	Decrease of 3.0 °C in the average T_a_
^52^	• Pavements with albedo 0.08 • Roofs with albedo 0.1 • Walls with albedo 0.25	• Pavements with albedo 0.2 • Roofs with albedo 0.3 • Walls with albedo 0.3	Decrease of 0.5 °C in the average T_a_
^53^	0.15	0.5	Decrease of 0.5 °C in the average T_a_
^54^	• Pavements with albedo 0.05 • Roofs with albedo 0.15	• Pavements with albedo 0.3 • Roofs with albedo 0.5	Decrease of 1.5 °C in the average T_a_
^55^	NA	Raising the albedo of rooftops by 0.25 and pavements by 0.15	Decrease of 0.11 to 0.53 °C in the average T_a_
^56^	NA	0.1 increase in the global albedo	Decrease of 0.3 to 0.5 °C in the average T_a_

The use of cooling surfaces that raise the albedo of roofs, walls, and pavements has proven effective in lowering T_s_ and lessening the severity of UHI in a number of cities (such as Beijing, Xi’an, and Rome)^12–15^. The cooling effect of mixing higher albedo surfaces; for example, high albedo roads with high albedo walls and roofs, has been investigated in recent studies^5,16^.

On the other hand, adding reflective materials to current urban surfaces provides a different strategy with a variety of uses, including white roofs and cool roads^17–19^. Compared to natural cooling systems, this technology requires less maintenance. Due to its ability to quickly control the energy balance and thermal climate in urban settings, the application of solar reflecting coatings to alter the albedo of urban surfaces has gained more attention^20,21^. Increasing road albedo lowers the T_s_ of the road in the middle to late afternoon, but it might not be a magic bullet for cooling cities ^22^. Urban roads with a higher albedo can reflect back more sunlight, which lowers the daytime temperatures of the surrounding air and the surface^23^. The impacts of high albedo surfaces, like walls, roofs, and roadways, have been thoroughly studied in a number of research. For instance, reflective pavements in California have been shown to reduce peak T_s_ by 4–6 °C during summer days^22^. This is in line with the findings of Elmagri et al., who observed that implementing reflective pavements in urban areas can lead to a decrease in T_s_ by up to 12 °C^24^. Another study highlighted that applying infrared reflective coatings to asphalt can reduce daytime T_s_ by up to 17 °C, depending on the material combination and pigment used^25^. In urban projects across Greece and Albania, implementing high-albedo pavements resulted in reductions of peak ambient temperatures by 1.2–2.1 °C, illustrating the broader applicability of such materials in diverse climates^26,27^. These findings are further supported by a summary of material types, albedo ranges, and their impact on T_s_ presented below in Table 2.

**Table 2:** Effect of increasing pavement’s albedo on surface temperature.

References	Pavements’ type	Albedo/properties	Results
^57^	Asphalt pavement	0.52	Decreases T_s_ by 4.0 °C at 12:00 PM in hot arid climate
^58^–^60^	Asphalt pavement	0.08 to 0.20	On a sunny day, lowers the temperature by 10 °C . Adding 0.1 albedo decreases the T_s_ by roughly 5–6 °C
^61,62^	Asphalt pavement	0.40 to 0.59	On a hot day, decreases T_s_ by roughly 15 °C at noon; maximally, decreased sensible heat by 200 W/m^2^
^63^	NA	0.09 to 0.66	Decrease T_s_ by 8 to 20 °C in hot summer
^25^	NA	0.31 to 0.59	Decrease T_s_ by a maximum of 12 °C
^62^	NA	0.46	Decrease 3.8 to 4.4 °C of Ts
^64^	Concrete pavements	0.80 to 0.90	In hot summer weather, decreases the daily T_s_ of a white concrete pavement by 4 °C and by 2 °C at night
^65^	Concrete pavement	0.76	Compared to a concrete pavement of the same colour, the daily T_s_ during hot summer situations decrease by 1 to 5 °C and by 1 °C at night
^66,67^	Concrete pavement	0.27 to 0.70	In comparison to concrete pavement of the same colour, the daily T_s_ under hot summer weather decrease by 2–10 °C
^68^	Asphalt pavement	0.46	5 °C decrease in daily temperatures when compared to concrete of the same colour
^69^	Asphalt pavement	0.50	Decrease in the pavement’s daily T_s_ by 8 to 15 °C and by 2 °C at night in comparison with conventional asphalt
^25^	Asphalt pavement	0.27 to 0.55	Decrease in the pavement’s daily T_s_ by 16 to 24 °C and by 2 °C at night in comparison with conventional asphalt
	Asphalt pavement	0.46 to 0.57	Decrease in the pavement’s daily T_s_ by 10.2 to 18.8 °C in comparison with conventional asphalt
^70^	Asphalt pavement	0.25 to 0.60	Decrease in the pavement’s daily T_s_ by 6.8 to 20 °C in comparison with conventional asphalt
^71^	Concrete pavement	• Coloured pavements have albedo of 0.51 to 0.78 • Colourless pavements have albedo of 0.71 to 0.81	Decrease in the pavement’s daily T_s_ by 5.4 to 10 °C in comparison with conventional asphalt
^57^	NA	• Albedo 0.52 • Emissivity 0.93	By using coated materials, T_s_ decreased to 4.4 °C
^39^	NA	0.35	Compared with conventional asphalt, reflective asphalt decreases T_s_ by 7.5 °C
^72^	NA	Reflectivity 60% Reflectivity 10%	Decreases Ts by 9 °C , has good waterproofing, and is age- and abrasion-resistant
^62^	NA	Reflectivity up to 81% Heat conductivity is low, of 0.252 W/(m*K). Maximum Emissivity of 0.83	T_s_ decreases to 17 °C
^73^	NA	Increase Reflectivity	10 ± 2.5 °C decreased on top and 10 ± 3 °C on the bottom
^44^	Application of photo catalytic asphalt on roadways	Infrared reflective cool paints with an albedo of 0.68 are used for colouring concrete pavements	Decrease of the pavements’ T_s_ to about 4.5 °C
^45^	NA	Infrared reflective cool paints with an albedo of 0.60 are used to colour concrete pavements	Decrease of the pavements’ T_s_ to about 12 °C

One possible approach for enhancing human thermal comfort in constructed environments is the use of higher albedo urban surfaces^28,29^. These surfaces efficiently lower T_s_ during particular daytime hours by reflecting solar radiation, improving outdoor thermal conditions during those times^30^. When high-albedo roads are used, the midday road T_s_ significantly drops, improving the thermal conditions for pedestrians^31^.

It was discovered that increasing the road’s and the wall’s solar reflectance from 0.2 to 0.7 and 0.5 to 0.8, respectively, can reduce the hot air by a maximum of 0.8 °C on a daily mean and up to 1.7 °C at its peak^32^. Some recent field experiments conducted in Los Angeles have shown that during the daytime hours of summer, the T_s_ of cool pavements can be 4–6 degrees Celsius less than that of ordinary asphalt pavements^22^.

However, the Mean Radiant Temperature (MRT) over a more reflective pavement surface than typical was 5.1 °C higher, according to data from a specifically designed instrument (MaRTy) developed by a study team at Arizona State University^32^.

### The role of surface albedo

On a scale of 0 to 1, the albedo measures a surface’s capacity to reflect back light. Albedo can be measured at two different scales in urban climatology: at the local-urban scale for the entire urban surface or at the scale of specific elements (such as roadways, façades, and roofs). Surface albedo, also known as Solar Reflectance (SR), is a measure of the reflecting power of individual surfaces. It is calculated by dividing the incident solar energy by the reflected solar radiation over a horizontal plane. Advanced ultra-white materials can have measured SR values as high as 0.95, whereas dark materials like asphalt can have measured SR value of 0.05^33^.

Urban roughness can increase solar absorption by up to 40%, as it traps solar reflections more effectively than flat planar surfaces^34–36^. This reduction in the reflecting power of urban surfaces plays a key role in amplifying the urban heat island effect^37^. In order to account for the combined influence of materials’ reflectivity and urban form occlusive properties, climatologists developed the concept of Urban Albedo (UA) to define the ability of the urban surface to bounce radiation back to the sky^33^.

At the top border of the layer of the urban canopy, which is the atmospheric layer that extends to just above roof level from ground level, UA is described as the ratio of incoming to reflected shortwave radiation. The usual range of variation of UA is lowered to roughly 0.2 to 0.4 as a result of the influence of urban geometry^33^.

Urban albedo can also be investigated at the micro level for certain urban canyons. At the level of street canyon eaves, where the roof plane meets the exterior walls, the ratio of reflected to incoming radiation is called the Urban Canyon Albedo (UCA)^33^.

Air pollution has been a major problem for cities for a long time. Cities are associated with massive power plants and the burning of fossil fuels, which are the main sources of air pollution, because of their high energy consumption. UHI tends to worsen air quality because heat accelerates chemical reactions in the atmosphere. According to Akbari et al.^9^, for every degree Celsius that the peak daily temperature increased above 22 degrees Celsius, the probability of urban pollution increased by 6%. Reflective materials change the ambient temperature throughout built terrain, which has a direct and indirect effect on urban air quality^7^.

The reflectivity of the materials determines how much solar energy is reflected off the surface. Reflective materials that have a higher solar reflectivity (albedo) tend to be whiter, absorb less light, and have a lower T_s_ throughout the day^38^. Numerous earlier research have reported this impact, the majority of which concentrated on the summer months^7,22,25,32,39^. For example, Middel et al. reported that cool pavements in Los Angeles exhibited T_s_ 4–6 °C lower than conventional asphalt during summer^22^, while Donthu et al. showed that increasing solar reflectance of road and wall surfaces led to reductions in T_a_ by up to 1.7 °C^32^. Similarly, reflective coatings and paints applied to asphalt and concrete have demonstrated reductions in daily T_s_ by 5–17 °C depending on the material and application technique^25,39^.

The study by Dimoudi et al.^40^ began with the notion that “according to their capacity to absorb, store, and reflect radiant energy, paved surfaces can help heat the air close to the surface when sunlight reaches it. This can have an impact on the urban microclimate.” This study compared the thermal behaviour of common building materials and cool materials in an urban center in Greece. According to the findings of Computational Fluid Dynamics (CFD) simulations, cool materials combined with other local mitigation strategies can lower the mean T_s_ of pavement and roadways by 6.5 °C. The microenvironment will be significantly impacted by this drop in temperature^41^.

In general, heat mitigation techniques involve altering the land cover to lessen solar absorption. It should be mentioned that a number of heat mitigation techniques have been studied and applied at different levels and in a variety of climates to lower the amount of energy used in cities^42^.

Erell has classified heat mitigation techniques into three categories: vegetation, cool pavement, and cool roofing. The cool pavements discussed in this study refer to the usage of light-coloured materials on the ground surface^43^. Depending on their albedo, these materials may reflect a significant amount of sunlight into the sky. In this sense, higher albedo surfaces lower sensible heat flux, which cools the system (urban area)^42^.

### Outdoor thermal comfort index: UTCI, PET, PMV

On hot days, cities suffer from severe thermal outdoor discomfort. Increasing surface reflectivity enhances the Outdoor Thermal Comfort (OTC) of an urban area by lowering both T_a_ and T_s_. A person’s thermal comfort is a somewhat arbitrary metric that is influenced by pertinent physiological, environmental, and other factors that impact how comfortable human bodies are. T_a_, Relative Humidity (RH), Wind Speed (WS), radiative exposure, ambient evaporative and sensible fluxes, and other environmental elements all affect human thermal comfort. Utilizing reflecting materials primarily alters the ambient temperature and radiation exposure, which changes the level of thermal comfort^7^.

According to a number of studies, T_a_, RH, WS, MRT, Predicted Mean Vote (PMV), and Physiologically Equivalent Temperature (PET) are the most useful metrics for evaluating OTC^74–77^.

The MRT is a crucial metric to measure thermal comfort in cities. WS and MRT are the main factors affecting thermal bioclimatic conditions, according to Schwarz et al.^78^ and Taha^79^.

Another scale that was introduced by Rizwanet al.^80^ is PET. PET is the T_a_ at which the human body’s energy distribution is balanced (in the absence of wind and sunlight) while keeping the same core and skin temperatures as within a challenging outdoor environment^81^.

Universal Thermal Climate Index (UTCI), is one of the indices for evaluating thermal comfort, it is derived from a thermo-physiological model of how people react to weather conditions, comprising the adaptation problem. The perceived temperature, or what the weather ‘feels like,’ is referred to UTCI^82^. It considers T_a_, WS, RH, and radiant temperature, which typically includes sun radiation. These inputs are used by UTCI in a human energy distribution model to provide a temperature value that represents the heat or cold strain that the body experiences^81^.

A study by Schrijvers et al.^83^ examined the impact of higher albedo materials on UTCI within a modelled street canyon in 2D. The research revealed that while high-albedo surfaces can reduce T_a_, they may also increase MRT due to enhanced reflection of solar radiation onto pedestrians and surrounding building constructions. This increase in MRT can offset the cooling benefits of reduced T_a_, leading to higher UTCI values and potentially decreased thermal comfort. The study concluded that the effect of albedo differences on UTCI is moderately small against the significant effects of shading, emphasizing the importance of considering urban geometry in thermal comfort.

Studies have shown that the efficacy of higher albedo materials in improving OTC varies depending on urban morphology, time of day, and specific climatic conditions. For instance, in narrow urban canyons, the increased reflection from high-albedo surfaces can lead to elevated MRT, potentially negating the cooling benefits. Conversely, in open areas, the reduction in T_a_ may outweigh the rise in radiant temperature, resulting in a net improvement in thermal comfort^24,33,83^. Middel et al. and Donthu et al. also observed that while reflective pavements reduce T_s_, they can simultaneously raise MRT due to intensified solar radiation reflection, especially during peak hours. These findings emphasize the need for a nuanced application of reflective strategies in different urban morphologies to optimize thermal comfort outcomes^22,32^.

Three variables relating to the outdoors thermal environment were chosen in order to asses people’s OTC. The surrounding thermal environment, human activity, clothes, and personal perceptions of heat in a given region are some of the aspects that impacts OTC^84^. The thermal comfort indicator Standard Effective Temperature (SET) and PET are frequently used to assess OTC^85^. By influencing the energy interaction between the human body and the environment, weather factors like T_a_, RH, radiation, and WS all have an effect on human thermal comfort^86^. Temperature is one of these factors that directly and significantly impacts human comfort in the urban microclimate. Therefore, three temperature indicators were chosen that are closely associated with outdoor human comfort: (1) UTCI, (2) pavement’s T_s_, and (3) T_a_ at 1.4 m height.

Higher surface albedo has been shown to have a valuable impact on T_s_ and UHI mitigation in a number of global locations. Nevertheless, an increasing amount of research indicates that improving paving’s solar reflectance has no influence on summertime OTC. This occurs because an urban environment exposes people to a variety of radiation sources that heat the body, including long-wave radiation from the sky and surrounding surfaces, reflected radiation from the horizontal and vertical surfaces, and incident solar radiation, both direct and diffuse. The MRT provides the clear effect on the radiative interaction with the body^24^.

Because of this, the MRT plays a key role in determining OTC indices such as PET. Because the increased reflected radiation may balance the decreased heat flux emitted from the horizontal surfaces, increasing solar reflectance could result in an increase in MRT. According to Salvati et al.^33^, this explains why reflective materials could negatively affect OTC. For instance, Donthu et al. reported that applying highly reflective coatings raised MRT by up to 5.1 °C compared to conventional pavements due to increased solar reflection onto pedestrians and surrounding vertical surfaces^32^. Supporting this, Błażejczyk et al. observed that high-albedo interventions, although successful in lowering T_s_, often resulted in elevated PET and UTCI readings by 1.5–3.0 °C, depending on the canyon aspect ratio and exposure^82^. Since this idea runs counter to the reviews compiled from other studies, this study will examine it and challenge it.

In hot-arid urban environments, reflective surfaces are widely used to reduce surface and air temperatures. However, an important trade-off has emerged in the literature: higher surface albedo may increase MRT due to enhanced solar reflection, particularly in compact urban forms such as narrow streets and high-aspect-ratio canyons. In such contexts, the increased reflectivity can intensify radiation exposure at pedestrian level, potentially counteracting the cooling benefits^33,87^. Therefore, the microclimatic effectiveness of reflective materials must be carefully examined within the spatial and morphological conditions of each urban setting.

This study addresses this concern by focusing on a low-density residential area in Cairo, where the urban form differs from typical high-density canyon-like streets. The selected case study features an H/W (height to width) ratio of slightly more than 1/2, which provides a more open configuration and greater sky exposure. Such urban settings are less prone to excessive shortwave reflection entrapment, making them more suitable for high-albedo surface interventions. By isolating the effects of surface reflectivity within this morphological context, the study investigates both the benefits and potential limitations of using reflective materials for outdoor thermal comfort enhancement.

### Urban morphology

The relationship between pavement albedo and urban morphology has a critical role in forming BED within urban environments. Pavement albedo refers to the reflectivity of pavement surfaces—higher albedo materials reflect more sunlight, potentially lowering surrounding T_a_ and reducing the cooling load for nearby buildings. However, the effectiveness of such interventions is not uniform across urban areas; it is significantly influenced by the morphological features of the built environment^6^.

Aspects of urban morphology that include building height, street width (or canyon aspect ratio), building density, and building construction length, determines how solar radiation interacts with surfaces and how heat is retained or dissipated within city spaces. For instance, in dense urban canyons with tall buildings and narrow streets (high H/W ratio), increased pavement albedo may have limited impact because the shading from buildings already reduces solar gain on the street level. Xu et al. found that in high-density zones, the reduction in BED due to increased pavement albedo was modest less than 2% annually while increasing pavement albedo resulted in T_a_ reduction of approximately 0.3–0.6 °C. Conversely, in areas with lower building heights and wider streets, reflective pavements can more effectively reduce ambient temperatures and influence BED. In medium-density areas, increasing albedo from 0.1 to 0.6 led to annual BED reductions of up to 8.5% and T_a_ reductions of up to 1.2 °C, while in low-density settings, effects varied—some zones even experienced slight increases in energy use during summer due to enhanced solar reflection contributing to indoor heat gains while the temperature drop reached 1.5–2.0 °C, depending on surface exposure and surrounding morphology^6^.

Two additional morphological metrics, Sky to View factor (S/V) and façade density further illuminate the correlation between urban form and thermal performance. A higher shape factor deduces more surface area is exposed to the external environment, which may increase a building’s sensitivity to outside temperature changes, while façade density affects how much of the building is exposed to solar radiation from the street. These parameters, when combined with albedo modifications, help explain why energy savings from reflective pavements can vary widely across different neighborhood types^6^.

The study of Xu et al.^6^ highlights this variation using Boston as a case study. In dense and medium-density areas, high pavement albedo generally led to decreased energy demand and carbon emissions due to lower cooling needs. However, in lower density zones, the energy savings were less consistent, some areas even experienced increase in energy use due to increase in cooling demands in summer outweighing heating savings in winter. This demonstrates that urban morphology must be carefully considered when deploying high-albedo materials, as their benefits are variable according to context and affected by the structural makeup of the surroundings.

Therefore, pavement albedo and urban morphology are deeply interconnected in their impact on urban energy performance. Effective urban planning should consider this interplay, using detailed morphological analysis to target reflective surface strategies where they can achieve the greatest net benefit.

Additionally, several researches examined how various urban morphologies such as vegetation, surface albedo, and adding water features affect OTC and provide advantages for residents. Four methods of heat mitigation in residential areas have been assessed by Lai et al.^88^: (a) applying cool surfaces; (b) altering urban geometry; (c) adding water features; and (d) placing vegetation. According to the findings, altering urban layout during the summer could result in the greatest improvement and the biggest decrease in T_a_ and PET. T_a_ was thus equal to 2.1 °C, and PET to 18.0 °C^89^.

The impact of shading characteristics on external thermal conditions in plateau climates has been investigated by Chen et al.^90^. It is determined that deciduous trees could increase OTC by 10.56 °C in the winter and 9.73 °C in the summer, respectively^89^.

Furthermore, Xu et al.^91^ measured the weather in Xi’an, China, to examine thermal comfort in various climate zones. The findings showed that tree shadowing effectively reduced heat stress by 10.1 °C in the summer and 15.5 °C in the winter. Beyond that, the UTCI is within the permissible range of 18.0–29.1 °C, with 23.1 °C^89^.

Meanwhile, street trees could enhance OTC conditions by 82% in an urban canyon with H/W = 0.5 to 0.7. Farhadi^92^ used Envi-met (V 4.3.0) for numerical simulation in order to evaluate the impact of vegetation, higher albedo surfaces, and building orientation on the UHI inside residential areas in Tehran, Iran. According to the findings, the T_a_ value dropped by 1.69 °C when the buildings’ orientation was changed. Meanwhile, a 10% increase in urban vegetation covering has resulted in the biggest gain in thermal comfort and a 9.36 °C drop in PET^89^.

Urban morphology plays a critical role in shaping microclimatic conditions in dense cities, particularly through its influence on wind patterns and air circulation. Street configurations, aspect ratios, and building setbacks can either facilitate or obstruct natural ventilation, directly impacting thermal comfort at pedestrian level. In hot-arid climates, where convective cooling is one of the few passive strategies available, interventions such as street widening and adjusting urban canyon geometry can significantly enhance wind flow and mitigate heat accumulation. Several studies have demonstrated that optimizing urban form can contribute to improved air movement, particularly during peak heat hours, thereby supporting passive cooling and thermal comfort enhancement in urban design strategies^93,94^.

This research gap exists as part of urban heat mitigation, particularly when considering strategies to reduce air convection without altering the context or introducing vegetation. In scenarios where the modification of the environment is restricted, changing the reflectivity of materials surfaces emerges as the only viable solution. By increasing the reflectivity of surfaces such as asphalt pavements, heat absorption can be reduced, potentially lowering the ambient temperature and decreasing the intensity of air convection. This approach focuses solely on the thermal properties of materials, without altering land use or adding vegetation. This research is primarily intended to explore how altering material reflectivity can effectively mitigate the movement of heated air, thus reducing the impact of convection currents and contributing to a more thermally stable environment in urban areas.

## Methodology

**Fig. 2:**
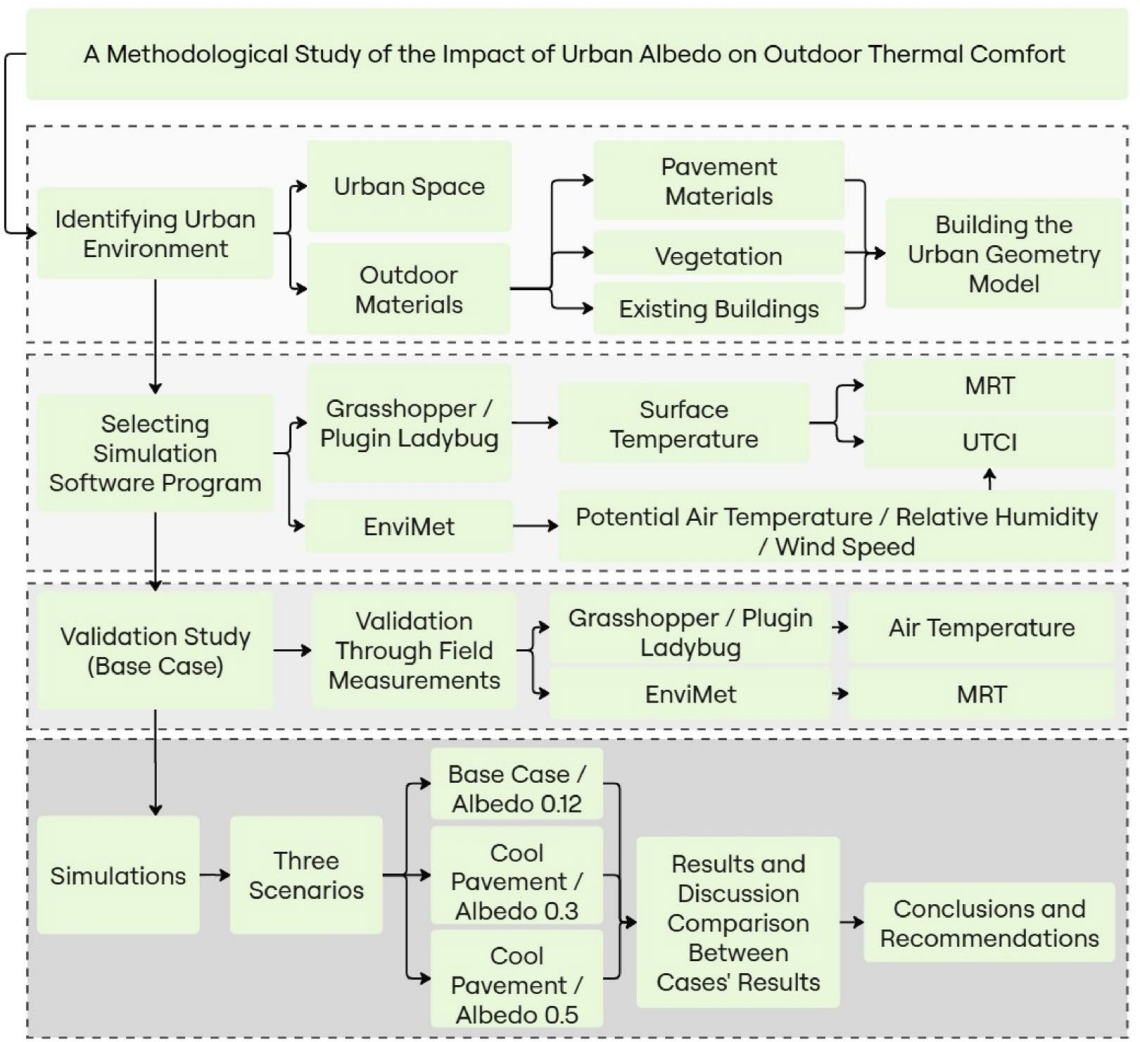
Methodology flowchart.

This section provides an overview of the analytical methods and tools employed in the research. It includes a parametric workflow for the dynamic thermal simulation of an urban space, implemented in the Grasshopper software and relying on its plug-in Ladybug as studied by Evola et al.^95^. In order to better understand how materials albedo affects environmental performance and urban T_s_, which has a direct impact on T_a_ in the context of urban sustainability. Starting from the base case, before new urban regulations, three different scenarios with varying values of the asphalt pavement’s reflectance were simulated by Grasshopper. The outcomes were evaluated against the base case model to illustrate their effects on street level microclimate and OTC. The overall structure of this analytical process including model development, simulation stages, and evaluation criteria is summarized below in Fig. 2, which illustrates the methodology flowchart used to guide the simulation and analysis steps.

## Research workflow

The methodological workflow employed in this study to investigate the impact of urban albedo on OTC is illustrated below in Fig. 2. The study commenced with field measurements conducted bi-hourly on July 29, 2024. Initial on-site assessments involved recording key urban features, including pavement materials, vegetation coverage, and the configuration of existing buildings, which were essential inputs for constructing an accurate urban geometry model. Following the data collection phase, the selection of simulation tools was carried out: Grasshopper with the Ladybug plug-in was chosen for simulating T_s_, MRT, and UTCI, while ENVI-met was employed to simulate T_a_, RH, and WS parameters.

To enhance the accuracy and depth of environmental simulations, outputs generated from ENVI-met were imported into Grasshopper. ENVI-met was selected for its ability to produce detailed microclimatic data, particularly at the pedestrian level, such as T_a_, MRT, and WS. The simulation domain was set using a cell size of 3 m, covering a spatial domain that balanced computational efficiency with resolution. However, due to the limitations of the free version of ENVI-met, which restricts the total area that can be modeled, the simulation was confined to a representative portion of the site.

To overcome this constraint and simulate larger areas, Grasshopper, through the Ladybug plugin, was utilized to extend the analysis at a macroclimatic scale. Environmental data from ENVI-met, including MRT, T_a_, and T_s_, were manually extracted and integrated into Ladybug components for advanced environmental evaluation. Additionally, Urban Weather Generator (UWG) was employed within Ladybug to simulate broader climatic interactions and urban morphology effects. This hybrid approach allowed the study to leverage ENVI-met’s high-resolution microclimate capabilities while taking advantage of Ladybug’s flexibility in large-scale and parametric analysis as demonstrated in previous studies that combined ENVI-met and Ladybug for multi-scale environmental modeling^5,96^.

A two-stage validation process was performed: first, by comparing simulated T_a_ from ENVI-met with field measurement readings, and second, by validating the simulated MRT from Grasshopper against the field observations. Upon successful validation, four models were developed: the first model represented the base case before the application of new urban regulations, characterized by a street width of 10 m; the subsequent three models simulated the post-regulation scenario with an expanded street width of 21 m. Among these, one model maintained the existing asphalt pavement albedo of 0.12, while the other two scenarios incorporated increased pavement albedo values of 0.30 and 0.50, respectively. The Results and Discussion section presents a detailed comparison of the simulation outcomes with findings from the literature, followed by the final Conclusion summarizing the study’s key insights.

According to Xu et al.^6^, albedo is the ratio of reflected shortwave radiation (wavelength 0.2 μm–3.0 μm) to the total amount of incoming shortwave radiation at the top of the atmosphere. The weighted mean albedo value in an urban recreation area was taken into account in this study.

To deduce those studies, a hybrid modeling framework was developed. This framework integrates the capabilities of several established tools, including the environmental and solar analysis plugin Ladybug for Grasshopper, ENVI-met 5.7.1 for microclimate simulation, and the 3D modeling platforms Rhinoceros (Rhino) and Grasshopper for urban form creation.

To achieve a comprehensive hybrid simulation, key microclimatic outputs from ENVI-met including T_a_, RH, and WS, were manually extracted from the ENVI-met output files and manually input into the Grasshopper platform via the Ladybug plugin. This integration was necessary because of file format incompatibility between ENVI-met and Ladybug.

To ensure temporal consistency, ENVI-Met’s hourly data were aligned with the simulation schedule used in Ladybug. This layered approach enabled the simulation of macro-scale environmental performance (e.g., MRT, UTCI) using micro-scale climatic inputs, bridging the operational strengths of both platforms. Figure 2 visually represents this workflow and illustrates the cross-platform data flow.

### Analytical methods

The author conducted a comprehensive observation and documentation of the selected urban area’s characteristics, focusing on its built environment and natural elements. This process included a detailed assessment of the architectural features of buildings, their materials, and height variations, as well as an analysis of street layouts, widths, and orientations to understand their influence on urban microclimate. Additionally, the presence, distribution, and density of vegetation, including trees, shrubs, and green spaces, were documented to evaluate their role in modifying thermal conditions and enhancing OTC. These observations provide essential contextual information for understanding the interactions between the urban form and its microclimatic conditions, forming the basis for further analysis.

The Sky View Factor (SVF) was calculated using the embedded algorithms available in the Ladybug plugin for Grasshopper. This component computes SVF based on 3D model geometry by analyzing the angular exposure of each analysis point to the sky hemisphere. The calculation considers the vertical and horizontal obstructions created by surrounding buildings and urban elements from a pedestrian-level perspective.

Façade density was determined by dividing the total vertical surface area of façades facing the street by the canyon’s cumulative length and average building height. All façade surfaces were assumed to be homogenous in material properties and thermal behaviour, as the study’s focus was limited to horizontal surface reflectivity. For modeling consistency, minor architectural details such as balconies, protrusions, and small-scale rooftop structures were excluded to streamline the computational process and emphasize dominant morphological features.

Grasshopper is an advanced parametric simulation program that accompanies Rhinoceros. An algorithmic sequence process that may evaluate many aspects of indoor and outdoor areas’ thermal performance is offered by the Grasshopper^96^. In order to identify and measure a variety of outdoor metrics, including the T_s_, MRT, and UTCI, this section provides the functional flow scripts and sequence structure that includes the Ladybug plugin. A parametric design workflow utilized to achieve the research goals is shown in Fig. 2. Buildings and outdoor spaces were among the first masses modelled in 3D-dimension out of the selected area. Secondly, the features of greenery and vegetation were developed. Additionally, a number of microclimatic environment simulations have been generated, including the Ts, MRT, and UTCI.

### Location and case study description

**Fig. 3:**
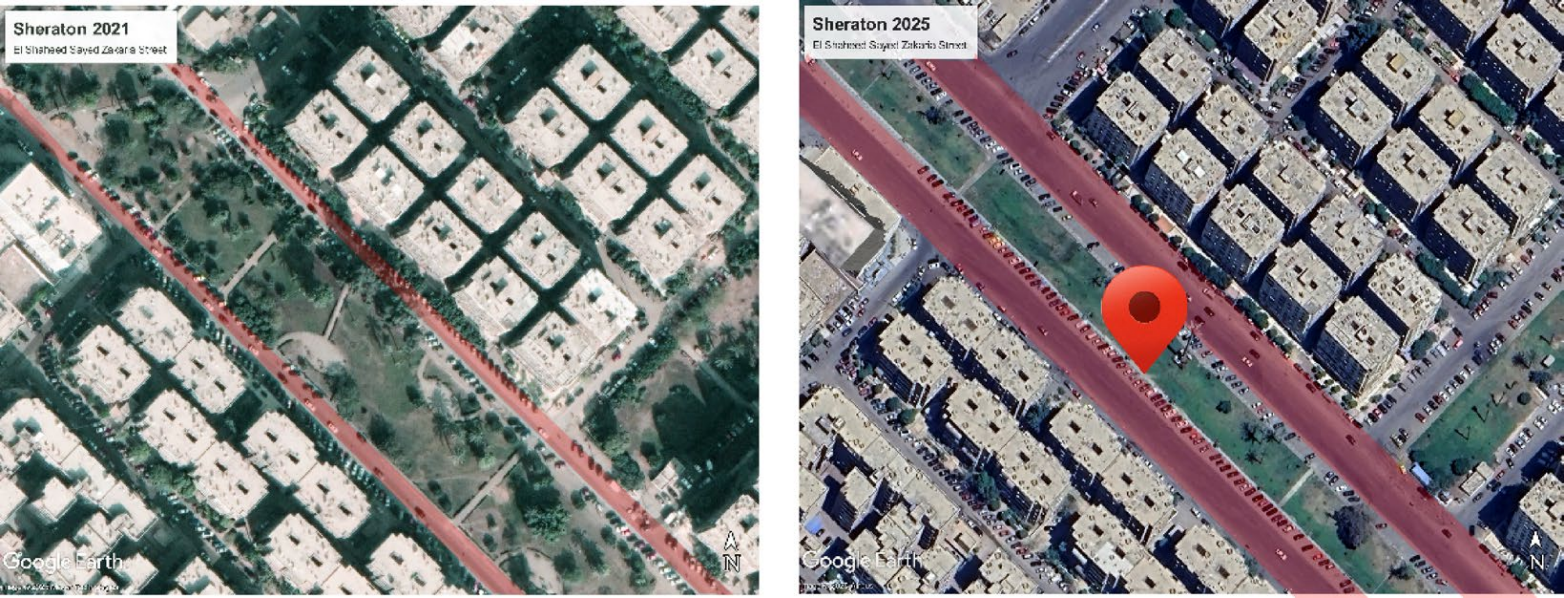
Ariel view for the selected area before and after new urban regulations.

The study intents to investigate the OTC in a pedestrian street in Sheraton, Cairo, Egypt that is well-known for having Feast-Festivals and other occasional celebrations which the new urban regulations were applied on and the street became more than triple the width while the vegetation were minimized consequently, as shown below the street development as highlighted in Fig. 3.

#### Urban morphology configuration

Urban morphology in this study was not addressed as a general concept but examined through a set of quantifiable spatial parameters that were systematically varied and tested across the simulation scenarios. These parameters are detailed in Table 3, which presents a comparative analysis of surface area components and urban form before and after the street transformation intervention.

**Table 3:** Comparison of surface area distribution and urban morphology before and after street development.

Surface area/Urban morphology	Greens in m^2^	Asphalt in m^2^	Pavements in m^2^	Building Density
Surface area in m^2^ while the Street width is 10 m	6155 m^2^	2731 m^2^	10,444 m^2^	8553 m^2^
Surface area in m^2^ while the Street width is 21 m	2626 m^2^	6260 m^2^	10,444 m^2^	8553 m^2^
Differences in area (m^2^)	3529 m^2^	3529 m^2^	0 m^2^	0 m^2^
Percentage of surface area while the Street width is 10 m	22.07%	9.79%	37.46%	30.67%
Percentage of surface area while the Street width is 21 m	9.42%	22.45%	37.46%	30.67%

Street width was explicitly modified as part of the urban intervention, representing a key variable in assessing the impact on T_a_, T_s_, and airflow at pedestrian height (1.4 m). The widening of the street created a more open configuration, allowing for increased ventilation and changes in thermal behaviour.

**Fig. 4:**
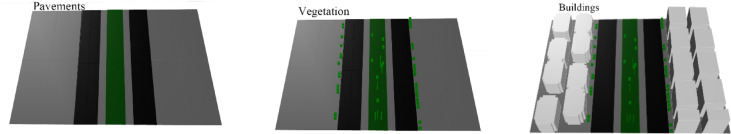
Modelling phases.

Although building heights did not change during the intervention, ranging from 18 m on the left side of the site in Fig. 4 to 27 m on the right, they were accounted for in the analysis as they influence the Sky View Factor (SVF) and the degree of radiative trapping. The spatial configuration between buildings remained constant, helping isolate the effects of other morphological variables.

Façade density was calculated as the ratio between the total vertical façade area and the total ground area within the study zone. This parameter was included to capture the degree of enclosure and its influence on thermal exchanges within the urban canyon.

Three different surface albedo values (0.12, 0.30, and 0.50) were tested to examine their interaction with morphological configurations. These albedo scenarios were applied across different urban forms to study how varying surface reflectivity impacts outdoor T_a_ and T_s_.

A major change highlighted in Table 3 is the substantial reduction in vegetation, which decreased from 6155 m^2^ to 2,626 m^2^, representing a loss of 3529 m^2^ and a decline in green area percentage from 22.07% to 9.42%. This vegetation loss was mirrored by a significant increase in asphalt surfaces, which expanded from 2731 m^2^ (9.79%) to 6260 m^2^ (22.45%). Pavement and building footprint remained unchanged, with pavement area fixed at 10,444 m^2^ (37.46%) and building footprint at 8553 m^2^ (30.67%). This intentional reduction of green spaces was tested to evaluate its thermal implications when combined with different albedo scenarios.

The data indicates that the changes focused on expanding roads and paved areas, as depicts in the increase in asphalt, while reducing green spaces. This shift likely disrupted the local microclimate, making the area warmer and less comfortable for people using the space. This alteration in surface composition, particularly the significant loss of vegetated zones, may lead to adverse environmental impacts if not mitigated by alternative cooling strategies.

### Simulation tools

A hybrid simulation approach was adopted in this study to enhance the accuracy and comprehensiveness of the environmental analysis. The process began with the use of ENVI-met, a three-dimensional microclimate simulation tool, to simulate T_a_, RH, and WS across the study area. These parameters, known for their spatial variability and critical influence on OTC, were extracted from ENVI-met due to its high-resolution capabilities in representing urban microclimatic conditions. Subsequently, the outputs from ENVI-met were integrated into Grasshopper—a parametric modeling environment—alongside the selected EPW weather file, which provided key climatic inputs such as direct normal radiation, diffuse horizontal radiation, and horizontal infrared radiation. The rural weather data were obtained from Climate One Building^97^, a reputable source that provides climate data specifically curated for building simulation worldwide. For the model input, Cairo, Egypt was selected as the study area^98^. This combination allowed for the simulation of MRT and the UTCI within Grasshopper using the Ladybug plug-in. The hybrid method ensured that the strengths of each software were utilized: ENVI-met provided detailed microclimate inputs, while Grasshopper enabled advanced thermal comfort modeling and greater flexibility in urban geometry analysis.

**Fig. 5:**
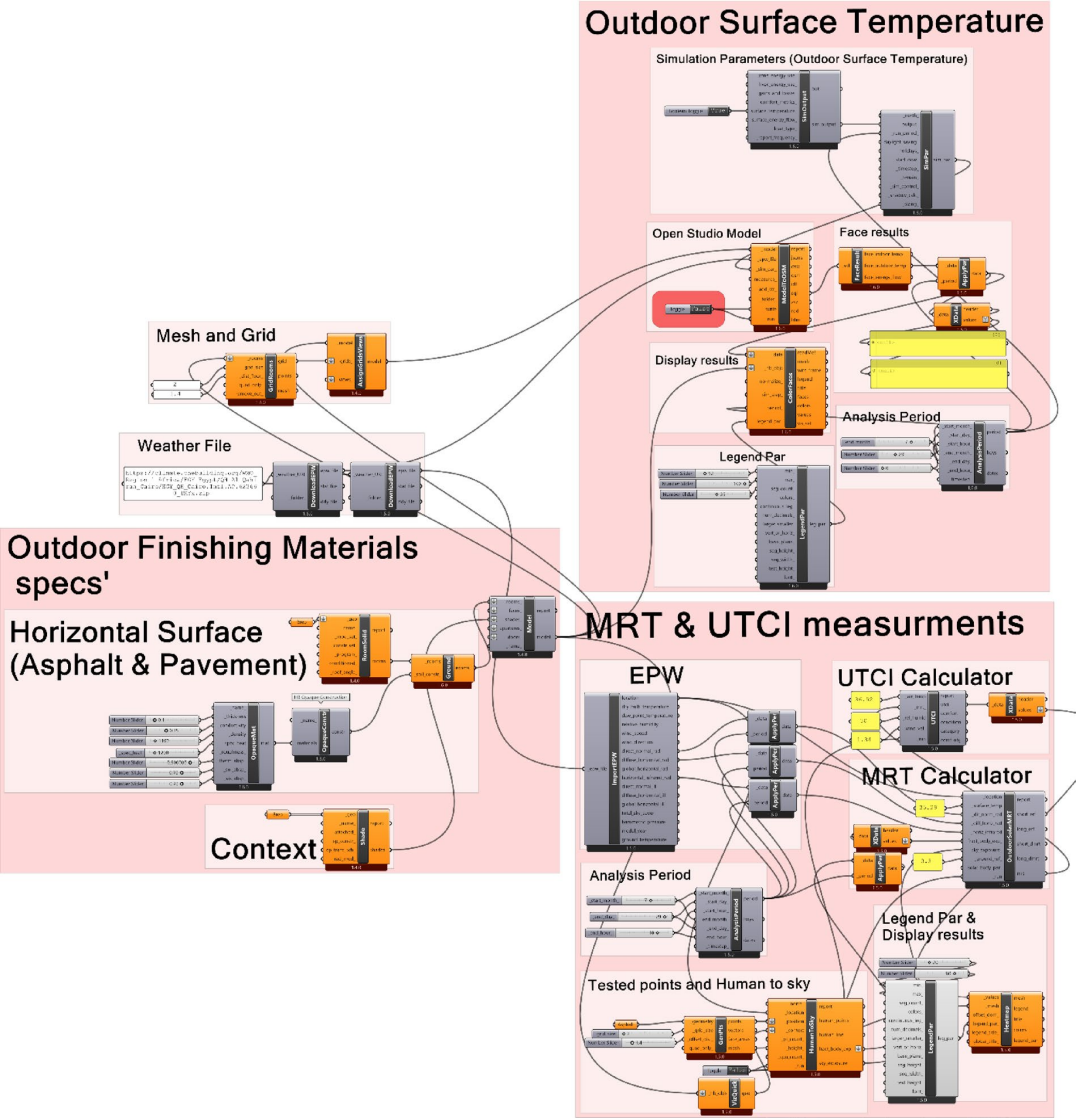
Grasshopper’s extensive parametric specification for outdoors surface temperature measurement, MRT, and UTCI.

The UTCI and MRT that match the real values shown in the validation section are deduced using an extensive parametric specification in Grasshopper. To measure UTCI and MRT the study followed Elmagri et al.^24^ method, the data input was converted to the Ladybug plug-in. Features like building typology, height, greenery percentage, surface albedo, and finishing materials are all included in the script. The correlation between the position of the human body and the sky was computed using the “LB Human to Sky Relationship” component. Two outcomes of the “LB Human to Sky Relationship” component are “Fraction Body Exposure and Sky Exposure,” which determine the sky value in the human body’s vision as well as the exposed human body to direct solar radiation. In order to determine MRT in the presence of surface reflectivity values for each element independently, all collected data was connected to “LB Outdoor Solar MRT.” The component uses a sky exposure approach to determine long wave radiant exchange with the sky and the ASHRAE-55^99^ to infer the impacts of solar shortwave. In order to determine the UTCI, the result MRT is directly linked to “LB UTCI Comfort (UTCI)”. Figure 5 below shows the parametric working flow in Grasshopper. Because this module was missing from the updated version, the previous iteration of Ladybug was used to measure T_s_. These inputs and data were linked to the “Honeybee Exported to Open Studio” component after the building, vegetation, topography, and testing period were determined. For the “Simulations Output” battery, which chooses which parameter to measure, this component primarily depends on the input of data. The outdoor T_s_ is the simulation data output by this component.

### Simulation process

On a typical hot day during the hottest summer month, July 29, 2024, the simulation was carried out. According to Abd Elraouf et al.^100^, July has the highest T_a_ and MRT and the greatest sunshine, with an average of 12.2 h per day. Since the early morning hours on summer days boost the somewhat warm thermal perception that impacts the MRT, which is the most influential component in OTC, the simulation will begin at 8:00 AM. To evaluate the data, this process lasts for ten hours until the early evening.

Several previous studies have identified July as the hottest month in Cairo, consistently recording the highest T_a_ and solar radiation levels, along with negligible rainfall^36,100^. These climatic characteristics make it an ideal reference point for evaluating outdoor thermal performance under extreme summer stress. Accordingly, 29 July 2024 was intentionally selected as the validation day in this study to align with peak summer conditions, the simulation will begin at 8:00 AM. To evaluate the data, this process lasts for ten hours until the early evening. This choice ensures that the reflective surface scenarios are tested under the most challenging environmental context, reinforcing the study’s relevance to heat mitigation in hot-arid climates.

To simulate realistic urban climatic conditions, a morphed EPW file derived from Climate One Building (COB) rural weather data was generated and imported into ENVI-met. This customized weather file accounted for urban microclimatic characteristics, particularly anthropogenic heat emissions from sources such as traffic, buildings, and surface heat storage. Adjustments were applied through ENVI-met’s boundary condition settings to reflect local heat forcing and ensure that the simulations represent the urban context rather than rural baselines. This approach enhances the reliability of the scenario analysis and aligns with methodologies used in recent studies that incorporated morphed or adjusted EPW datasets for urban-scale environmental modeling^101,102^.

### Validation and verification of the model

Sensitivity analysis was used for model validation and verification for the purpose of evaluating the significance of the simulation patterns and results. After that, field-measured data and the main modelling outputs were compared. It was established how sensitive MRT and T_s_ were to variations in surface albedos at various points, either between buildings or one-sided buildings. Using calibrated instruments for monitoring T_a_, RH, and WS, all of which were used to compute T_s_ for MRT simulation—the predicted outcomes were contrasted with the actual measurements made in the chosen area^103^.

The field measurements of T_a_, RH, and WS were adopted in order to verify the ENVI-met and LB&HB models. Based on their placement in an outdoor setting, these factors helped to provide thermal comfort for humans, hence they were employed for validation.

The process of collecting microclimatic data including T_a_, RH, and WS using specialized equipment at various urban locations. Both exposed and shaded regions were used for the fieldwork to ensure a comprehensive understanding of the site’s thermal conditions. These measurements provided essential input for validating simulation models and analyzing the impact of surface reflectivity and urban morphology on OTC.

The simulation findings were contrasted with measurements made on July 29, 2024, in the same area. T_a_, RH, and WS were among the variables gathered across the field investigation. These were computed using Eq. 1 that is shown below that used the data to find MRT, and the outcomes of the simulations were then contrasted with the data. Following Elwy et al., the same equation was used as referenced in^104^, which is based on ISO 7726 and incorporates air velocity, globe temperature, emissivity, and globe diameter to ensure accurate estimation under outdoor conditions. In this study, the globe thermometer was used to measure MRT. It was modeled as a hollow copper sphere with a 150 mm diameter (D) and an emissivity (εℊ) of 0.95, following standard thermal comfort specifications^105^.

1 $$\sqrt[4]{{\left( {Tg + 273} \right)^{4} + \frac{{1.1*10^{8} *V^{0.6} }}{{\varepsilon_{g} *D^{0.4} }}}}*\left( {T_{g} - T_{a} } \right) - 273$$

Equation 1. MRT calculation.

It was deduced that the cool pavement surface had an albedo of between 0.3 and 0.5. Five distinct times of the day were used for the comparisons. Tables 4 and 5 show the five times of the day where simulation results and measurements seem to coincide well. The overall discrepancy between measurements and models is less than 3.0%.

**Table 4:** The values of field measurements and simulated data for MRT.

Time	Simulated	Measured
8:00	57.25	58.59
10:00	69.75	68.86
12:00	72.25	70.22
14:00	78.50	76.81
16:00	71.00	69.55

**Table 5:** The values of field measurements and simulated data for Air Temperature.

Time	Simulated	Measured
8:00	31.53	34.20
10:00	36.46	36.00
12:00	39.48	38.50
14:00	42.34	41.15
16:00	41.34	38.60

**Fig. 6:**
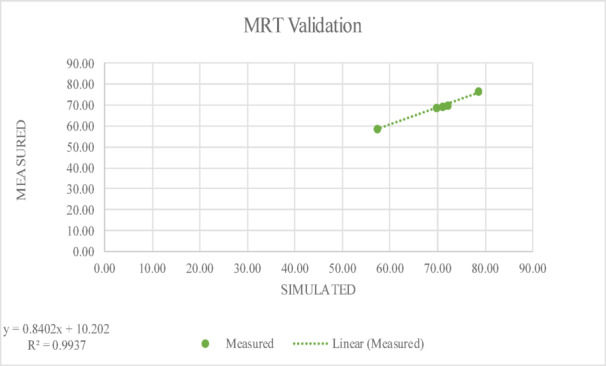
The R-squared (R^2^) value between measured and simulated data for MRT.

**Fig. 7:**
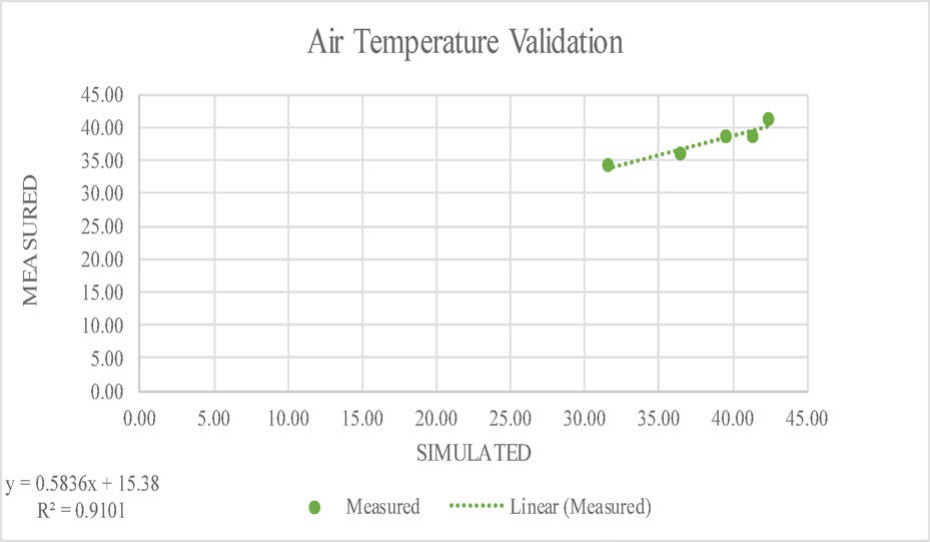
The R-squared (R^2^) value between measured and simulated data T_a_.

The R-squared (R^2^) value is a statistical metric that indicates the degree of correlation between simulated results and field measurements. In this study, the R^2^ value is above 0.9 as shown below in Figs. 6 and 7 which demonstrates a strong agreement between the measured and simulated data, deducing that the model effectively captures real-world conditions. A high R^2^ value indicates that the majority of the variability in the field measurements is accurately represented by the simulation, verifying the modelling approach’s validity and dependability.

In Fig. 6, which presents MRT validation, a strong linear correlation is observed between simulated and measured values, with a coefficient of determination (R^2^) of 0.9297. Similarly, Fig. 7 shows the validation of T_a_ values simulated using ENVI-met with an R^2^ of 0.9101, which also deduces a very good fit between simulated and measured T_a_. Although the model performed well overall, with high R^2^ values for MRT and T_a_, some small differences were noticed during midday hours and early night. These differences might be due to quick weather changes like wind or clouds that are hard to capture in the simulation. Still, the results stayed within a reasonable and acceptable range. Together, these figures confirm the accuracy of the hybrid simulation approach employed in this study, supporting the credibility of the subsequent scenario analysis. These results are in line with previous validation studies that reported similarly high R^2^ values between simulated and measured data^106,107^.

Three instruments were utilized to collect field measurements: the first measured T_a_, the second recorded RH, and the third assessed WS. These instruments will be discussed in detail in the following paragraphs.

To verify the accuracy of the field measurements for MRT, a strict validation process was carried out in alignment with internationally approved standards, specifically ISO 7726:1998, “Ergonomics of the thermal environment: Instruments for measuring physical quantities”^108^. Before conducting any on-site measurements, all instruments underwent calibration and testing in a controlled environmental laboratory to ensure that each device met the required accuracy specifications.

**Fig. 8:**
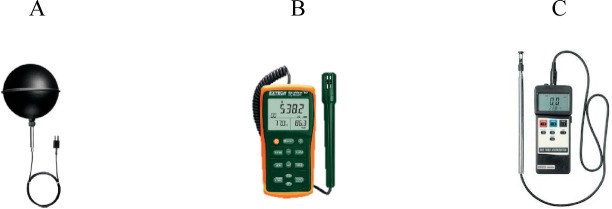
The instruments used in data collection for validation.

The Testo 0602 0743 Globe Probe (thermocouple type K) shown below in Fig. 8A, is a specialized instrument used for measuring T_a_ with a focus on assessing OTC and indoor environments. This probe features a black globe sensor, which allows it to capture not only the ambient T_a_ but also the effects of radiant heat from surrounding surfaces. Its thermocouple type K technology ensures fast response times and reliable measurements, making it a valuable tool for heat stress analysis, microclimate studies, and UHI research^105^.

The Extech EasyView Indoor Air Quality Meter/Data Logger shown below in Fig. 8B, is a versatile instrument designed for monitoring and recording key indoor environmental parameters. It is commonly used to measure carbon dioxide (CO₂) levels, temperature, humidity, and dew point^109^.

A hot-wire thermos-anemometer shown below in Fig. 8C, is a precision instrument used to measure air velocity and temperature in various environmental and industrial applications. It operates by heating a thin wire to a constant temperature and detecting changes in electrical resistance as airflow cools the wire, allowing for highly accurate and rapid measurements. Due to its high sensitivity and fast response time, it is especially useful for studying turbulent airflows, thermal comfort, and aerodynamic performance in both indoor and outdoor environments^110^.

All measurements were taken at points that were fully exposed to direct solar radiation, without any obstruction from nearby buildings, vehicles, or trees—in other words, representing the worst-case scenario in terms of solar exposure. Measurements were recorded at a height of 1.4 m, corresponding to pedestrian level, to accurately capture thermal conditions experienced by individuals.

### Albedo values selection

The selected albedo values of 0.12, 0.30, and 0.50 represent a realistic and literature-supported range of surface reflectivity, reflecting both materials commonly used in Cairo and established research benchmarks. The 0.12 value corresponds to low-reflective dark asphalt surfaces typically applied in urban roadways^111^. The 0.30 value reflects medium-reflectivity concrete surfaces or surface-treated pavements found in local applications^112^. The 0.50 value represents highly reflective surfaces such as white concrete or engineered cool pavements, and aligns with thresholds commonly defined for effective heat mitigation^113^. This selected range supports comparative analysis between existing urban conditions and potential material interventions intended to reduce T_s_ and T_a_ in hot-arid settings.

## Results

### Urban morphology’s effect on air temperature

**Fig. 9:**
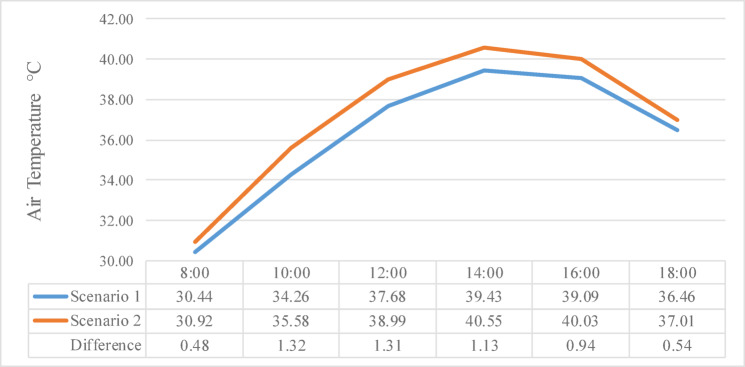
Air temperature comparison before and after new urban regulations.

Figure 9 illustrates the effect of increased surface area specifically through street widening on T_a_ variation throughout the day, from 8:00 AM to 6:00 PM. The chart compares hourly T_a_ values for Scenario 1, which represents the base case with a street width of 10 m, and Scenario 2, where the street was widened to 21 m. The data shows that T_a_ increased after the widening, with noticeable differences, this phenomenon is due to the air convection resulting from the expanded surface area, which promotes greater heat absorption and exchange with the surrounding environment.

At 8:00 AM, T_a_ in Scenario 2 was 30.92 °C, recording a 0.48 °C increase compared to Scenario 1, where the temperature was 30.44 °C. This difference increased by 10:00 AM, with Scenario 2 reaching 36.29 °C compared to 35.58 °C in Scenario 1, resulting in a 1.32 °C rise. At 12:00 PM, the temperature difference remained similarly elevated, with Scenario 2 measuring 38.99 °C and Scenario 1 at 37.68 °C, reflecting a 1.31 °C increase. By 2:00 PM, temperatures increased to 40.55 °C in Scenario 2 compared to 39.43 °C in Scenario 1, narrowing the difference slightly to 1.13 °C.

The peak daily difference occurred at 4:00 PM, where Scenario 2 recorded 40.03 °C compared to 39.09 °C in Scenario 1, marking a 0.94 °C increase. Even in the late afternoon at 6:00 PM, the temperature in Scenario 2 remained higher, reaching 37.01 °C against 36.46 °C in Scenario 1, resulting in a 0.54 °C difference. These consistent temperature increases throughout the day reflect the thermal impact of the street widening intervention. The rise in T_a_ suggests that the increased exposed pavement surface and the corresponding reduction in shading contributed to greater heat accumulation during the day.

However, after the introduction of new regulations potentially involving increased building density, modified land use, or changes in material reflectance, T_a_ exhibited a measurable rise. This shift deduces that the new urban policies may have altered the local microclimate, potentially reducing natural ventilation, increasing heat retention, or modifying surface radiation properties.

### Pavement albedo’s effect on air temperature

**Fig. 10:**
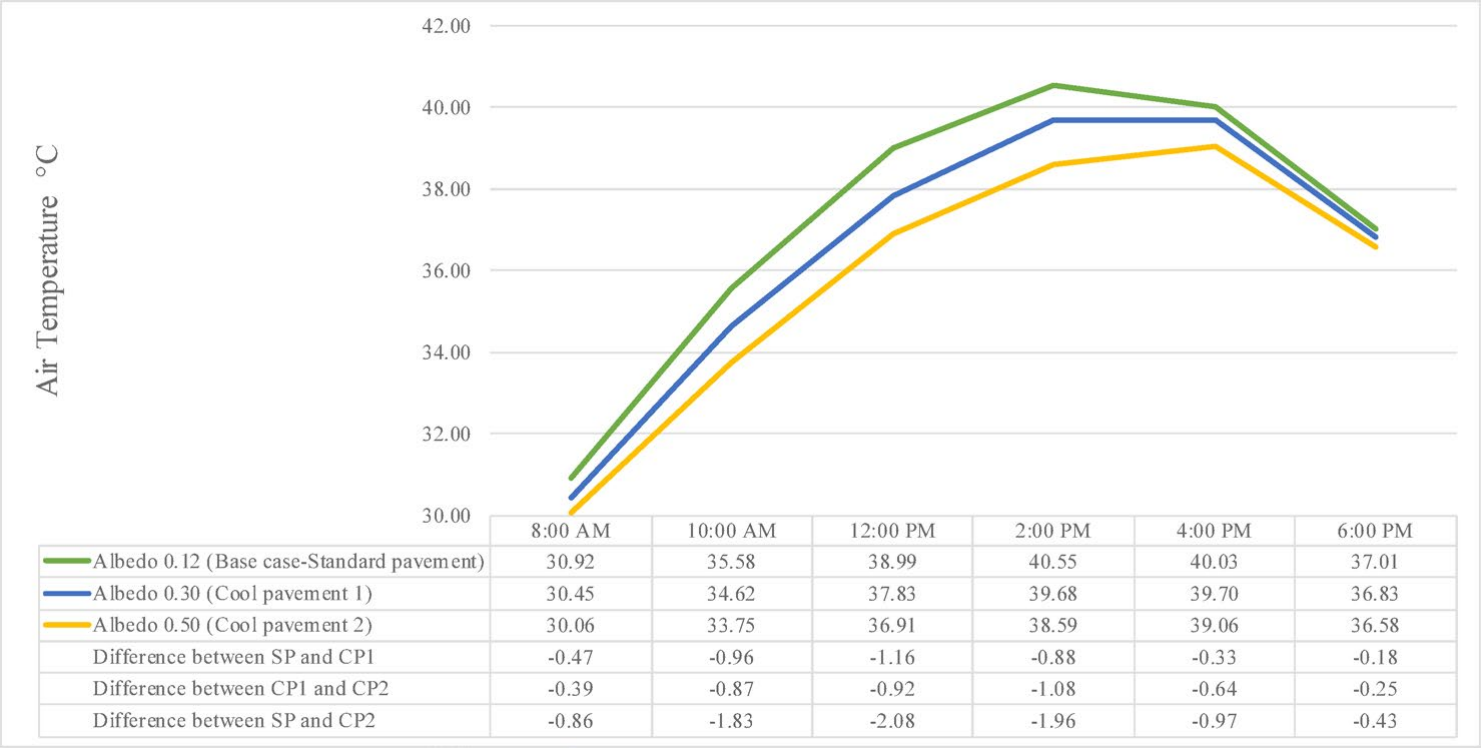
Air temperature comparison between different surface albedo.

While increasing the albedo’s value from 0.12 (base case-standard pavement) to 0.30 (cool pavement 1) and 0.50 (cool pavement 2), Fig. 10 illustrates a clear reduction in T_a_ throughout the day, from 8:00 AM to 6:00 PM. The results reveal a consistent inverse relationship between surface albedo and T_a_, where higher albedo values correspond to lower ambient temperatures across all time intervals.

At 8:00 AM, T_a_ in the Standard Pavement (SP) was 30.92 °C, recording a 0.47 °C increase compared to Cool Pavement 1 (CP1) at 30.45 °C, and a 0.86 °C increase compared to Cool Pavement 2 (CP2) at 30.06 °C. By 10:00 AM, the temperatures increased to 35.58 °C for SP, 34.62 °C for CP1, and 33.75 °C for CP2, resulting in differences of 0.96 °C and 1.83 °C between SP and CP1, and SP and CP2, respectively. At 12:00 PM, this pattern continued, with SP reaching 38.99 °C, while CP1 and CP2 measured 37.83 °C and 36.91 °C, leading to widened differences of 1.16 °C and 2.08 °C, respectively, highlighting the intensifying cooling effect of more reflective pavements under stronger solar radiation.

At the daily peak at 2:00 PM, SP recorded 40.55 °C, compared to 39.68 °C for CP1 and 38.59 °C for CP2, showing temperature differences of 0.87 °C and 1.96 °C, respectively. This cooling pattern persisted at 4:00 PM, where SP measured 40.03 °C, exceeding CP1 by 0.33 °C at 39.70 °C, and CP2 by 0.97 °C at 39.06 °C. Even by 6:00 PM, SP remained warmer at 37.01 °C, while CP1 and CP2 recorded 36.83 °C and 36.58 °C, resulting in smaller yet consistent differences of 0.18 °C and 0.43 °C, respectively. These results confirm that CP2, representing the highest surface reflectivity (albedo 0.50), consistently produced the lowest T_a_ across the day, emphasizing its effectiveness in mitigating urban heat buildup and improving outdoor thermal comfort.

### Urban morphology’s effect on surface temperature

This section examines the impact of street widening on T_s_ by comparing the two morphological scenarios mentioned earlier. Both scenarios were simulated under identical environmental conditions to isolate the effect of increased surface area.

**Fig. 11:**
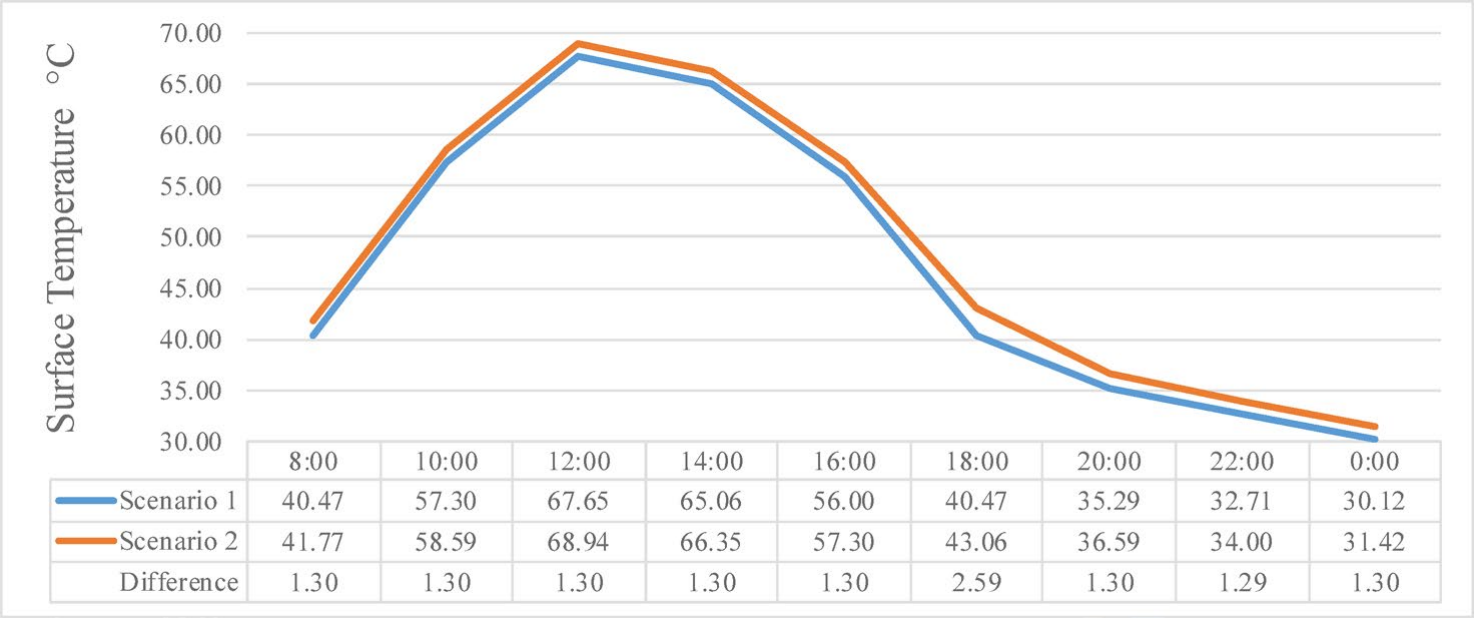
Comparison between surface temperature before and after new urban regulations.

As illustrated in Fig. 11, Scenario 2 consistently exhibited higher T_s_ than Scenario 1 throughout the day. At 8:00 AM, the pavement temperature in Scenario 1 was 40.47 °C, while in Scenario 2 it was 41.77 °C, recording a difference of 1.30 °C. This difference remained relatively steady in the morning, with a 1.29 °C increase recorded at both 10:00 AM and 12:00 PM. At 10:00 AM, T_s_ were 57.30 °C in Scenario 1 and 58.59 °C in Scenario 2, while at 12:00 PM, they measured 67.65 °C and 68.94 °C, respectively.

At 2:00 PM, the difference persisted at 1.29 °C, with Scenario 1 registering 65.06 °C and Scenario 2 at 66.35 °C, indicating the sustained thermal impact of additional exposed pavement. At 4:00 PM, the T_s_ in Scenario 2 was again 1.30 °C higher, with temperatures of 56.00 °C for Scenario 1 and 57.30 °C for Scenario 2. While by 6:00 PM, the difference increased notably to 2.59 °C, as Scenario 1 cooled to 40.47 °C while Scenario 2 remained warmer at 43.06 °C, reflecting the accumulated heat stored in the larger paved area.

These results clearly demonstrate that increasing the street width led to measurable increases in T_s_ at all-time intervals. The consistent temperature differentials emphasize the influence of urban morphology on thermal behaviour, particularly the role of surface area in modulating solar exposure and heat retention within the urban fabric.

### Pavement albedo’s effect on surface temperature

Road T_s_ decrease as road albedo increases. Roads that have a high albedo reflect more sunlight into the atmosphere, which lowers temperatures by reducing the amount of energy absorbed by the road surface^114^. The cooling impact of raising road albedo, however, varies spatially throughout Cairo and works best in places with a moderate building density.

**Fig. 12:**
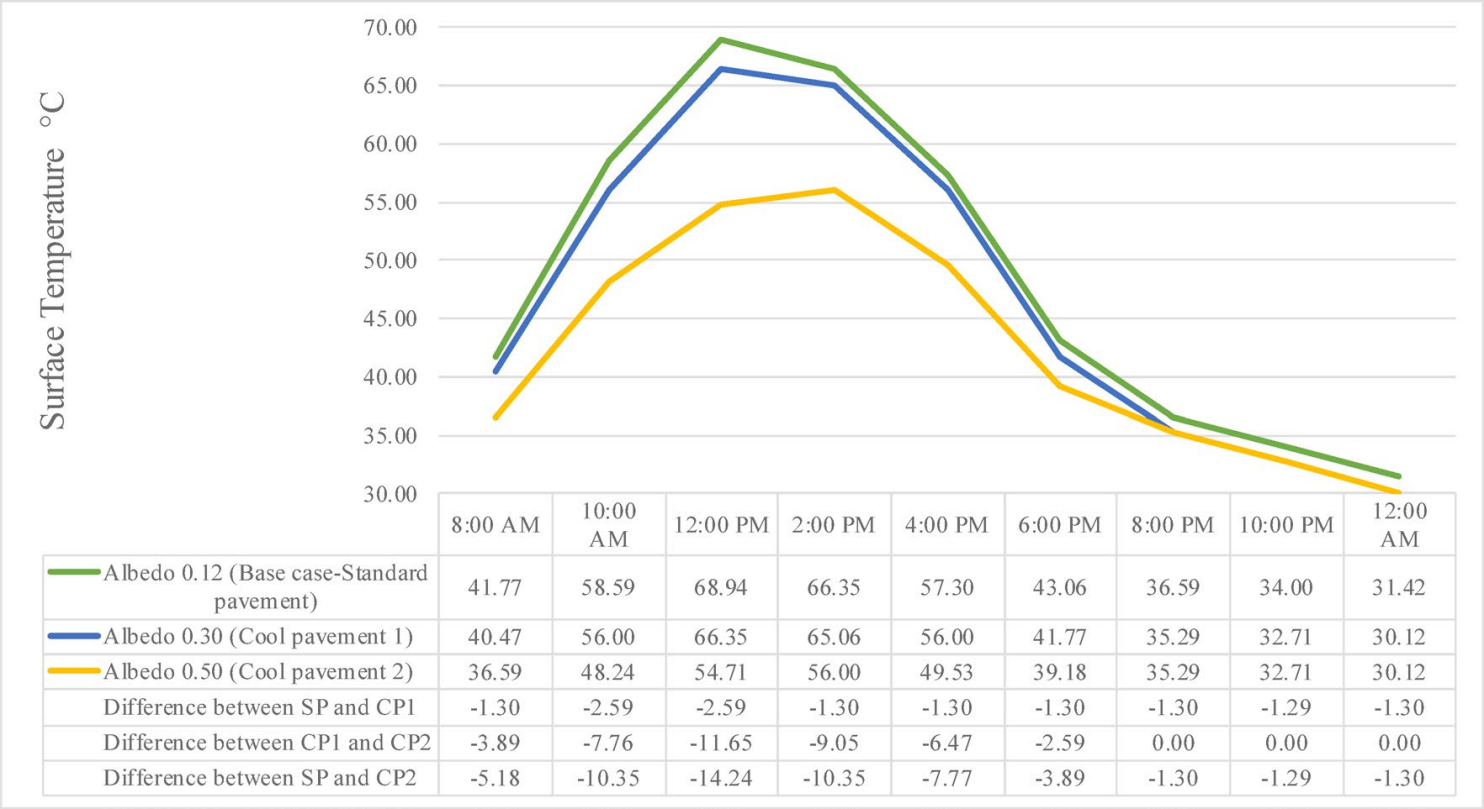
The correlation of asphalt’s surface temperature between different albedo values.

Figure 12 presents the variation in asphalt T_s_ over a 24-h period for the three pavement types: SP with albedo 0.12, CP1 with albedo 0.30, and CP2 with albedo 0.50. The results clearly demonstrate the cooling effect of increased surface reflectivity throughout the day.

At 8:00 AM, the T_s_ for SP was 41.77 °C, recording a 1.30 °C higher value compared to CP1 at 40.47 °C, and 5.18 °C higher than CP2 at 36.59 °C. By 10:00 AM, these differences expanded, with SP measuring 58.59 °C, CP1 at 56.00 °C, and CP2 at 48.24 °C, resulting in differences of 2.59 °C between SP and CP1 and 10.35 °C between SP and CP2. At the midday peak (12:00 PM), SP reached 68.94 °C, exceeding CP1 at 66.35 °C by 2.59 °C and CP2 at 54.70 °C by a notable 14.24 °C. At 2:00 PM, the temperatures were 66.35 °C for SP, 65.06 °C for CP1, and 56.00 °C for CP2, corresponding to differences of 1.29 °C (SP against CP1) and 10.35 °C (SP against CP2).

As the afternoon progressed, the cooling advantage of CP surfaces persisted. At 4:00 PM, SP recorded 57.30 °C, CP1 recorded 56.00 °C, and CP2 registered 49.53 °C, resulting in SP being 1.30 °C warmer than CP1 and 7.77 °C warmer than CP2. While by 6:00 PM, T_s_ dropped to 43.06 °C for SP, 41.77 °C for CP1, and 39.18 °C for CP2, decreasing the differences to 1.29 °C (SP against CP1) and 3.88 °C (SP against CP2). During the nighttime period at 12:00 AM, SP remained at 31.42 °C, CP1 at 30.12 °C, and CP2 at 30.12 °C, resulting in SP being 1.30 °C warmer than both CP1 and CP2.

To better understand the diurnal behaviour of reflective materials, T_s_ reductions were analyzed separately for peak daytime hours and nighttime periods across the three tested albedo values (0.12, 0.30, and 0.50). As shown in Fig. 12, the maximum daytime reduction reached 12.94°C for albedo 0.50, while the nighttime reduction was more modest, peaking at approximately 2.3°C. This variation highlights that albedo-driven cooling is most effective during daytime, when solar radiation is at its peak. However, its impact at night remains limited, especially in outdoor environments where radiant cooling and urban ventilation dynamics become more dominant. Therefore, complementary strategies such as urban shading, vegetation, or enhanced nighttime airflow should be considered to optimize outdoor thermal comfort throughout the full diurnal cycle.

Overall, CP2 consistently maintained the lowest T_s_ throughout the 24-h period. It recorded a maximum T_s_ of 56.00 °C at 2:00 PM, which is 12.94 °C lower than SP at its peak of 68.94 °C at 12:00 PM. These findings confirm that increasing pavement albedo especially to 0.50, significantly reduces surface heat accumulation, thereby contributing to the mitigation of the UHI effect and improving thermal conditions in urban settings.

### MRT and UTCI

**Fig. 13:**
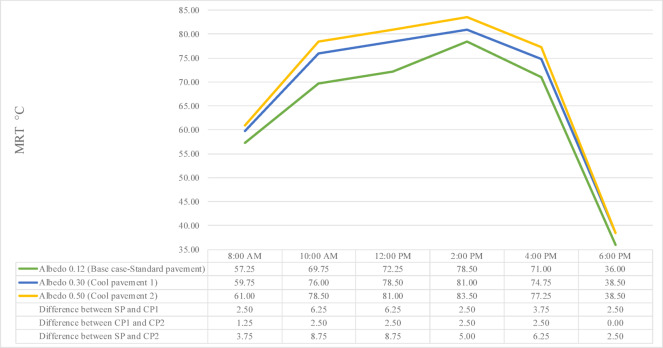
The correlation between MRT during the day and albedo values**.**

**Fig. 14:**
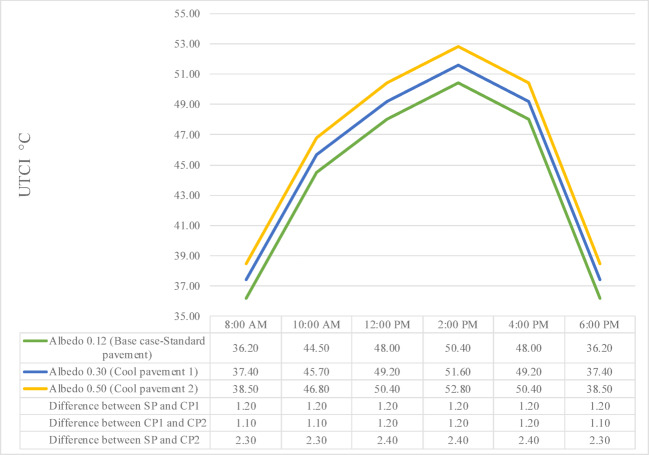
The correlation between UTCI during the day and albedo values.

The impacts on MRT and UTCI derived from the previously discussed case study will serve to assess the efficacy of the cool pavement (CP) strategy compared to the conventional or standard pavement (SP) approach. A detailed mapping of MRT and UTCI will be conducted across different microenvironments, including beneath trees, between buildings, and over pavement surfaces, following the evaluation of T_a_ and T_s_. For example, Figs. 13 and 14 present the MRT and UTCI distributions, respectively, across various zones and time periods.

#### Pavement albedo’s effect on MRT

Geometries and urban surroundings have a significant impact on MRT. MRT is regarded as one of the most crucial factors in assessing OTC and is very responsive to various urban design techniques. The hourly MRT for each of the three Albedo settings is displayed in Fig. 13.

Figure 13 illustrates the variation in MRT across different surface albedo values for SP, CP1, and CP2 throughout the day from 8:00 AM to 6:00 PM. MRT is a critical factor in OTC, and changes in surface albedo significantly influence this parameter.

At 8:00 AM, the MRT values are relatively close for all three surfaces. The lowest MRT is observed with the SP surface at 57.25 °C, while CP1 records 59.75 °C and CP2 records 61.00 °C. The MRT in SP is 2.5 °C lower than CP1 and 3.75 °C lower than CP2. As albedo increases, the MRT also slightly increases across the surfaces. This pattern continues at 10:00 AM, where the MRT rises notably to 69.03 °C for SP, 76.00 °C for CP1, and 77.78 °C for CP2. Here, the difference becomes more distinct, with SP showing MRT values 6.25 °C lower than CP1 and 8.75 °C lower than CP2, as higher albedo surfaces reflect more shortwave radiation, contributing to increased MRT.

By 12:00 PM, MRT levels peak for all surfaces: 72.25 °C for SP, 78.50 °C for CP1, and 81.00 °C for CP2. The differences remain consistent, with SP being 6.25 °C cooler than CP1 and 8.75 °C cooler than CP2. The influence of surface reflectivity becomes clearer, as MRT increases progressively with higher albedo. This pattern strengthens further at 2:00 PM, where the MRT values reach their highest levels: 78.00 °C for SP, 80.50 °C for CP1, and 83.00 °C for CP2. At this peak, SP shows an MRT 2.5 °C lower than CP1 and 5.0 °C lower than CP2, highlighting the thermal impact of surface reflectivity during peak solar exposure.

At 4:00 PM, a slight decline begins, with MRT values dropping to 71.00 °C for SP, 74.75 °C for CP1, and 77.25 °C for CP2. SP remains 3.75 °C cooler than CP1 and 6.25 °C cooler than CP2, although the sun’s intensity begins to wane. Higher albedo surfaces still exhibit elevated MRT levels compared to lower ones. By 6:00 PM, MRT plummets for all surfaces as solar radiation decreases sharply. SP records 36.90 °C, CP1 records 38.00 °C, and CP2 records 39.40 °C, maintaining smaller differences where SP remains 1.10 °C cooler than CP1 and 2.5 °C cooler than CP2, leveling off the earlier disparities.

Overall, the data demonstrates a clear correlation between surface albedo and MRT, where higher albedo consistently leads to higher MRT throughout the day, particularly during peak solar hours. This phenomenon indicates that while increasing albedo can be beneficial for reducing T_s_ due to more reflected radiation, it may inadvertently raise the MRT, potentially affecting pedestrian thermal comfort in exposed urban areas.

#### Pavement albedo’s effect on UTCI

Figure 14 depicts the UTCI for a street surface with varying albedo values 0.12, 0.30, and 0.50 that stands for SP, CP1, and CP2 respectively, over the course of a day, from 8:00 AM to 6:00 PM. UTCI is a key metric used to assess OTC, as it reflects the combined effects of T_a_, RH, radiation, and WS on the human thermal experience. The graph highlights how surface reflectivity influences UTCI throughout the day.

At 8:00 AM, UTCI values are lowest across all surfaces as solar radiation is still moderate. SP records the lowest UTCI at 36.2 °C, while CP1 records 37.4 °C and CP2 records 38.5 °C, resulting in CP1 showing an increase of 1.2 °C and CP2 an increase of 2.3 °C compared to SP. This indicates that in early morning hours, higher surface reflectivity does not immediately reduce perceived thermal stress but still results in slightly higher discomfort. By 10:00 AM, the UTCI values increase significantly across all surfaces. SP rises to 44.5 °C, CP1 to 45.7 °C, and CP2 to 46.8 °C, with CP1 exhibiting a UTCI approximately 1.2 °C higher than SP and CP2 showing an even greater increase of 2.3 °C. This pattern indicates that higher surface reflectivity correlates with increased thermal load during morning hours.

At 12:00 PM, UTCI peaks are observed, reaching 48.0 °C for SP, 49.2 °C for CP1, and 50.4 °C for CP2. The differences remain consistent, with CP1 recording a UTCI about 1.2 °C higher than SP, and CP2 about 2.4 °C higher. These figures suggest that reflective surfaces amplify the perceived heat stress around midday when solar exposure peaks. At 2:00 PM, when UTCI reaches maximum daily values, SP records 50.4 °C, CP1 rises to 51.6 °C, and CP2 increases to 52.8 °C. The differences persist, with CP1 approximately 1.2 °C higher than SP and CP2 about 2.4 °C higher, reinforcing the notion that higher albedo materials contribute to greater thermal stress under intense sunlight.

By 4:00 PM, although UTCI values start to decrease, SP records 48.0 °C, CP1 records 49.2 °C, and CP2 records 50.4 °C, maintaining a pattern where CP1 is about 1.2 °C above SP and CP2 is about 2.4 °C higher. The elevated thermal stress linked to higher reflectivity is still evident even as solar radiation decreases. At 6:00 PM, UTCI values drop substantially as solar exposure diminishes. SP shows a UTCI of 36.2 °C, CP1 records 37.4 °C, and CP2 records 38.5 °C, with CP1 maintaining a UTCI approximately 1.2 °C higher than SP and CP2 showing an increase of 2.3 °C. This closes the daily thermal comfort cycle, highlighting that throughout the day, increased albedo consistently results in higher perceived heat stress.

The diagram shows that contrary to the common assumption that higher albedo always enhances thermal comfort, in this case, higher surface reflectivity leads to higher UTCI values throughout the day. This is likely due to increased shortwave reflection adding to the radiant heat experienced by pedestrians. Therefore, while increasing albedo may cool T_s_, its effect on overall thermal comfort, as measured by UTCI, may not always be beneficial in exposed urban settings.

## Discussion and conclusion

The results of this study clearly demonstrate that raising road albedo leads to a significant reduction in T_a_ throughout the day, which aligns closely with previous findings in the literature. For instance, as shown in Fig. 10, streets with higher albedo surfaces (0.50) consistently exhibited lower T_a_ than those with albedo 0.12. These findings are in agreement with Donthu et al.^32^, who observed that increasing road reflectivity could reduce T_a_ by a maximum of 0.8 °C on a daily mean and up to 1.7 °C during peak hours. Similarly, Ferrari et al.^23^ and Elmagri et al.^24^ documented how reflective surfaces help mitigate urban heat accumulation, particularly during high solar exposure periods. In the context of hot arid climate, where dense morphology exacerbates heat retention, these cooling effects from high-albedo materials could be critical for reducing daytime heat stress. Likewise, another research found that an increase of 0.1 in road surface albedo can cause a T_a_ drop of more than 3 °C on a highway in China’s Hunan Province^115^.

The simulations indicated that increasing surface albedo from 0.12 to 0.50 decreased T_s_ by an average of 6.2 °C, with the highest reductions occurring during midday. These findings are consistent with observational studies from Los Angeles, where cool pavements reduced summer T_s_ by 4–6 °C^22^. Further alignment is seen with field data referenced in Refs.^25^ and^39^, where albedo treatments led to reductions of up to 17 °C depending on material type and application method. Also, it was discovered that high-albedo roads in the Phoenix metropolitan region had little effect on wall temperatures in densely populated residential neighborhoods and limited cooling effects on air and road T_s_ at 2 m^116^. According to some recent field tests carried out in Los Angeles, cool pavements can have T_s_ that are 4–6 °C cooler than regular asphalt surfaces throughout the summer^22^. This underscores the practicality of high-albedo strategies in climates like hot-arid, especially when vegetation or shading is limited.

While albedo improved T_s_ and T_a_, the results revealed that both MRT and UTCI increased with higher albedo, a trend confirmed in Figs. 13 and 14. This paradox reflects similar concerns raised in studies by Yang et al.^7^ and Donthu et al.^32^, where high-reflectance surfaces elevated MRT by 5.1 °C due to increased shortwave radiation being bounced onto the human body and surrounding urban elements. This reflected radiation, while reducing conduction-based heat absorption, increases radiative load, leading to higher thermal discomfort as represented by UTCI. The results indicated an average increase of 2.35 °C in UTCI between albedo 0.12 and 0.50, echoing findings from^83^, where elevated MRT offset cooling gains from lower T_a_ in narrow urban canyons. Likewise, Błażejczyk et al.^82^ concluded that areas with higher albedo surfaces exhibit higher levels of thermal stress, according to research measuring urban thermal comfort using UTCI. This highlights the potential unintended effect of surface reflectivity on OTC and the complexity of mitigating UHIs.

UTCI values in this study exceeded 50 °C during peak summer hours, indicating extreme heat stress conditions that align with findings from recent studies in similar hot-arid climates^24,117^. These results were highlighted to reflect the growing impact of climate change, which is intensifying outdoor thermal discomfort and pushing UTCI values beyond historically observed thresholds in the region. The occurrence of such high values is consistent with previous records of outdoor thermal stress in comparable environments and emphasizes the need to reassess urban design practices under future climate projections.

While the application of high-albedo materials demonstrated effective thermal reduction at the surface and air levels, the study also acknowledges a potential trade-off: increased exposure to reflected radiation may aggravate human thermal stress in certain urban configurations. This highlights the importance of evaluating both microclimatic outcomes and human-centric comfort parameters when proposing surface interventions. Future implementations should be complemented with shading elements or context-specific design measures to balance the benefits of albedo enhancement with pedestrian-level comfort, as supported by previous studies Huang et al. and Salvati et al.^33,87^.

Urban morphology exerts a critical moderating role in the thermal outcomes of reflective surfaces, as reinforced by the case study and emphasized in the literature. As Xu et al.^6^ noted, the cooling benefits of reflective pavements vary across urban typologies, with dense, shaded zones responding differently than wide, exposed streets. The study area after widening experienced heightened heat accumulation as depicted in Figs. 9 and 11, reinforcing the idea that reflective strategies must be integrated with urban geometry considerations. This was further supported by the documented reduction in greenery from 22 to 9%, which likely compounded the radiative load on pedestrians. This also aligns with findings in Refs.^89,91^ that reducing vegetation cover and increasing built surfaces lead to thermal degradation in urban areas.

This paper demonstrates the proven effectiveness of increasing road surface albedo in residential areas as a means to enhance OTC, addressing the environmental challenges observed in Cairo City streets. The study responds to recent urban regulations that have been implemented without prior assessment of OTC between building constructions, which has contributed to a reduction in thermal comfort hours and limited the usability of outdoor spaces for various resident activities. According to a number of simulations and tools the study’s findings determine the ideal asphalt albedo setting that alters and enhances outdoor human comfort in residential areas during the summer.

In an urban setting, MRT, UTCI, T_a_ and T_s_ calculations provide a thorough understanding of how well different pavement finishes affected local thermal processes. The process was developed using the software Grasshopper that operates compatibly with Rhinoceros. Ladybug plugin was used in functional flow scripts to evaluate a range of outdoor thermal metrics. Field data in the same location validated simulations conducted in Sheraton Heliopolis neighborhood.

For the purpose of modeling the effects of high-albedo surfaces in diverse urban settings, the author used Cairo as a case study for hot arid climate. The results show that raising urban surface albedo is a beneficial strategy for enhancing the OTC, but it necessitates a thorough analysis of urban morphology and factors related to the OTC. In neighborhoods with low- to mid-rise buildings and wide streets free of shading elements or buildings constructions that could throw shadows on roadways, high albedo urban pavements show remarkable usefulness in promoting the release of reflected shortwave radiation out of the urban boundary layer.

While the study focused on reflective horizontal surfaces as a standalone heat mitigation strategy, the findings also highlight the potential benefits of integrating additional cooling elements. In particular, the reduction of green areas in the study site was associated with a measurable increase in T_s_ and T_a_. This underscores the importance of vegetation in regulating urban microclimate. Accordingly, combining reflective materials with vegetation-based solutions, such as tree planting or vertical greenery systems, could enhance overall thermal performance. These integrated approaches may provide both shading and evaporative cooling, making them especially valuable in hot-arid urban environments where traditional green spaces are limited.

However, while the implementation of high-albedo materials has proven effective in reducing T_s_ and T_a_, it may also introduce a potential drawback and increased exposure to reflected solar radiation, which can elevate thermal stress on pedestrians, particularly in dense urban configurations such as narrow streets or urban canyons. This trade-off highlights the necessity of considering not only the microclimatic benefits of surface cooling but also the human-centric implications of increased reflectivity. To ensure more thermally comfortable environments, future designs should integrate context-appropriate shading solutions alongside reflective materials. These concerns have been emphasized in recent research, including Huang et al. and Salvati et al.^33,87^, which underscores the importance of a balanced and integrated approach to urban heat mitigation.

Urban planners and policy-makers play a critical role in translating the findings of this study into effective, context-sensitive interventions. While this research was limited to horizontal surface reflectivity, future urban design strategies could incorporate shading structures that are tailored to the street’s specific geometry and constraints. Planners could suggest suitable canopy types that fit the spatial conditions without conflicting with existing regulations, while also increasing shaded zones to counteract the elevated MRT caused by higher surface albedo. In parallel, selecting tree species and vegetation types with strong shading capacity and evapotranspiration potential would help absorb excess radiant heat and improve outdoor thermal comfort in hot-arid urban contexts.

## Research limitations

This research is limited in scope to the investigation of horizontal urban surfaces (specifically pavements) without any modifications to vertical or overhead elements. The study did not incorporate shading structures, such as pergolas, wooden canopies, or other artificial elements, due to municipal restrictions and the intended focus on surface reflectivity as an isolated mitigation strategy.

Additionally, no modifications were made to building façades. Treatments that could have enhanced solar reflectance or reduced heat absorption on building envelopes were excluded to maintain the study’s emphasis on ground-level materials only.

Another limitation involves the role of vegetation and evapotranspiration. While these are significant factors in urban thermal regulation, their impact was not simulated in this study. This decision was justified by the actual condition of the case study area, which contains very limited green spaces and an extremely low number of trees, making the contribution of transpiration negligible.

Furthermore, the study was constrained by stringent height restrictions imposed by the proximity of two major airports near the site. As a result, interventions involving building height adjustments or vertical shading elements could not be explored. The study area also represents a low-density residential zone, characterized by a height-to-width (H/W) ratio of slightly more than 1/2, which may limit the generalizability of the findings to denser urban canyons.

In addition, while increasing surface albedo can reduce Ts and Ta, it may also result in elevated human thermal stress due to greater levels of reflected solar radiation reaching pedestrians. This effect can be particularly pronounced in compact urban forms, such as street canyons or narrow pathways, where multiple reflections intensify shortwave radiation exposure at body level. As noted by Huang et al. and Salvati et al.^33,87^, such reflections may offset the cooling benefits of reflective surfaces if not properly mitigated through complementary shading or vegetation strategies.

## Future studies

Future research could explore the application of this methodology to different types of asphalt materials, particularly those with varied thermal and reflective properties. Examining how alternative surface compositions influence T_a_ and T_s_ could provide a broader understanding of material performance under different climatic loads. Additionally, extending the simulation period beyond the summer season to cover a full annual cycle would offer insights into seasonal variation and long-term thermal behaviour. Such year-round analyses would enhance the generalizability of the findings and support more comprehensive urban heat mitigation strategies.

Moreover, future studies could also incorporate vegetation-based solutions, such as tree planting, vertical greenery systems, or green facades, in combination with reflective materials. These strategies may offer a synergistic effect by simultaneously reducing T_s_ and improving outdoor thermal comfort through evapotranspiration and shading. Investigating such combinations would provide more integrated and effective urban cooling approaches, particularly in areas where space for horizontal greening is limited.

Furthermore, the long-term performance of high-albedo materials, particularly considering real-world factors such as surface soiling, material degradation, and maintenance requirements should be addressed. While high-reflectivity pavements show promising short-term benefits in reducing T_s_ and T_a_, their thermal effectiveness may decrease over time due to accumulated dust, pollutants, and organic deposits, especially in arid and dusty urban environments like Cairo. In addition, durability and wear resistance under traffic and weathering conditions remain critical challenges that may affect their cost-effectiveness and sustainability. Therefore, future research should include field-based monitoring and aging simulations to evaluate reflectivity loss and maintenance cycles over time^118^.

Additionally, future research should investigate the combined application of high-albedo surfaces with other passive cooling strategies, such as vegetation, shading structures, and cool roofs, to achieve more effective and balanced thermal comfort outcomes. While this study focused solely on horizontal surface reflectivity, integrating reflective pavements with urban trees and canopies could enhance both radiative and convective cooling, particularly under hot-arid conditions. Studies such as^10,119,120^ have shown that multi-strategy approaches outperform isolated interventions in mitigating urban heat and improving outdoor thermal comfort.

In addition, upcoming research should explore the potential of emerging reflective technologies, including radiative-cooling surfaces, retro-reflective pavements, and phosphorescent coatings. These innovative materials are designed to enhance surface cooling efficiency beyond what is achievable with conventional high-albedo treatments, while also addressing known limitations such as degradation, maintenance, and declining performance over time. Their application in urban contexts may lead to more durable and efficient heat mitigation solutions. Recent studies, such as^118,121–125^ have demonstrated the promising thermal performance of such materials in both experimental and field settings.

## Data Availability

Data availability: The data that support the findings of this study are available on request from the corresponding author.

## References

[1] Sen, S. & Roesler, J. Aging albedo model for asphalt pavement surfaces. *J. Clean. Prod.* **117**, 169–175. 10.1016/j.jclepro.2016.01.019 (2016).

[2] Gago, E. J., Roldan, J., Pacheco-Torres, R. & Ordóñez, J. The city and urban heat islands: A review of strategies to mitigate adverse effects. *Renew. Sustain. Energy Rev.* **25**, 749–758. 10.1016/j.rser.2013.05.057 (2013).

[3] Heaviside, C., Macintyre, H. & Vardoulakis, S. The urban heat island: Implications for health in a changing environment. *Curr. Environ. Heal. Rep.* **4**(3), 296–305. 10.1007/s40572-017-0150-3 (2017). 10.1007/s40572-017-0150-3 28695487

[4] Piracha, A. & Chaudhary, M. T. Urban air pollution, urban heat island and human health: A review of the literature. *Sustainability* **14**(15), 9234. 10.3390/su14159234 (2022).

[5] Liu, Y. et al. Impacts of high-albedo urban surfaces on outdoor thermal environment across morphological contexts: A case of Tianjin, China. *Sustain. Cities Soc.* **100**, 105038. 10.1016/j.scs.2023.105038 (2024).

[6] Xu, X., AzariJafari, H., Gregory, J., Norford, L. & Kirchain, R. An integrated model for quantifying the impacts of pavement albedo and urban morphology on building energy demand. *Energy Build.*10.1016/j.enbuild.2020.109759 (2020).

[7] Yang, J., Wang, Z. H. & Kaloush, K. E. Environmental impacts of reflective materials: Is high albedo a ‘silver bullet’ for mitigating urban heat island?. *Renew. Sustain. Energy Rev.* **47**, 830–843. 10.1016/j.rser.2015.03.092 (2015).

[8] Li, D., Bou-Zeid, E. & Oppenheimer, M. The effectiveness of cool and green roofs as urban heat island mitigation strategies. *Environ. Res. Lett.* **9**(5), 055002. 10.1088/1748-9326/9/5/055002 (2014).

[9] Akbari, H., Menon, S. & Rosenfeld, A. Global cooling: Increasing world-wide urban albedos to offset CO_2_. *Clim. Change* **94**(3), 275–286. 10.1007/s10584-008-9515-9 (2009).

[10] Science, U., Abdelwahab, R. A., Fekry, A. A., El, R. & Hamed, D. The effective landscape design parameters with high reflective hardscapes: Guidelines for optimizing human thermal comfort in outdoor spaces by design—A case on hot arid climate weather. *Comput. Urban Sci.*10.1007/s43762-025-00186-w (2025).

[11] Yang, W., Wong, N. H. & Li, C. Q. Effect of street design on outdoor thermal comfort in an urban street in Singapore. *J. Urban Plann. Dev.* **142**(1), 05015003. 10.1061/(asce)up.1943-5444.0000285 (2016).

[12] Battista, G. & Pastore, E. M. Using cool pavements to mitigate urban temperatures in a case study of Rome (Italy). *Energy Proc.* **113**, 98–103. 10.1016/j.egypro.2017.04.027 (2017).

[13] Zhu, Z., Zhou, D., Wang, Y., Ma, D. & Meng, X. Assessment of urban surface and canopy cooling strategies in high-rise residential communities. *J. Clean. Prod.* **288**, 125599. 10.1016/j.jclepro.2020.125599 (2021).

[14] Zhang, L., Fukuda, H. & Liu, Z. The value of cool roof as a strategy to mitigate urban heat island effect: A contingent valuation approach. *J. Clean. Prod.* **228**, 770–777. 10.1016/j.jclepro.2019.04.338 (2019).

[15] De Luca, F. Advances in climatic form finding in architecture and urban design. *J. Archit. Educ.* **49**(2), 79–91 (2010).

[16] Morini, E., Touchaei, A. G., Castellani, B., Rossi, F. & Cotana, F. The impact of albedo increase to mitigate the urban heat island in Terni (Italy) using the WRF model. *Sustainability* **8**(10), 1–14. 10.3390/su8100999 (2016).

[17] Akbari, H. & Matthews, H. D. Global cooling updates: Reflective roofs and pavements. *Energy Build.* **55**, 2–6. 10.1016/j.enbuild.2012.02.055 (2012).

[18] Levinson, R. et al. A novel technique for the production of cool colored concrete tile and asphalt shingle roofing products. *Sol. Energy Mater. Sol. Cells* **94**(6), 946–954. 10.1016/j.solmat.2009.12.012 (2010).

[19] Rosado, P. J. Evaluating cool impervious surfaces: Application to an energy-efficient residential roof and to city pavements. U*niversity California, Berkeley ProQuest Diss. Theses*. (2016).

[20] Carlosena, L., Ruiz-Pardo, Á., Rodríguez-Jara, E. Á. & Santamouris, M. Worldwide potential of emissive materials based radiative cooling technologies to mitigate urban overheating. *Build. Environ.* **243**, 110694. 10.1016/j.buildenv.2023.110694 (2023).

[21] Wang, J., Liu, S., Meng, X., Gao, W. & Yuan, J. Application of retro-reflective materials in urban buildings: A comprehensive review. *Energy Build.* **247**, 111137. 10.1016/j.enbuild.2021.111137 (2021).

[22] Middel, A., Turner, V. K., Schneider, F. A., Zhang, Y. & Stiller, M. Solar reflective pavements—A policy panacea to heat mitigation?. *Environ. Res. Lett.* **15**(6), 064016. 10.1088/1748-9326/ab87d4 (2020).

[23] Ferrari, A., Kubilay, A., Derome, D. & Carmeliet, J. The use of permeable and reflective pavements as a potential strategy for urban heat island mitigation. *Urban Clim.* **31**(Mar 2019), 100534. 10.1016/j.uclim.2019.100534 (2020).

[24] Elmagri, H., Kamel, T. M. & Ozer, H. Assessment of the effectiveness of cool pavements on outdoor thermal environment in urban areas. *Build. Environ.* **266**, 112095 (2024).

[25] Synnefa, A. et al. Experimental testing of cool colored thin layer asphalt and estimation of its potential to improve the urban microclimate. *Build. Environ.* **46**(1), 38–44. 10.1016/j.buildenv.2010.06.014 (2011).

[26] Santamouris, M. et al. Improving the microclimate in a dense urban area using experimental and theoretical techniques—The case of Marousi, Athens. *Int. J. Ventil.* **11**(1), 1–16. 10.1080/14733315.2012.11683966 (2012).

[27] Fintikakis, N., Gaitani, N., Santamouris, M., Assimakopoulos, M. & Assimakopoulos, D. N. Bioclimatic design of open public spaces in the historic centre of Tirana, Albania. *Sustain. Cities Soc.* **1**(1), 54–62. 10.1016/j.scs.2010.12.001 (2011).

[28] Hardin, A. W. & Vanos, J. K. The influence of surface type on the absorbed radiation by a human under hot, dry conditions. *Int. J. Biometeorol.* **62**(1), 43–56. 10.1007/s00484-017-1357-6 (2018). 28477222 10.1007/s00484-017-1357-6

[29] Tabatabaei, S. S. & Fayaz, R. The effect of facade materials and coatings on urban heat island mitigation and outdoor thermal comfort in hot semi-arid climate. *Build. Environ.* **243**, 45–67 (2023).

[30] Back, Y., Bach, P. M., Jasper-Tönnies, A., Rauch, W. & Kleidorfer, M. A rapid fine-scale approach to modelling urban bioclimatic conditions. *Sci. Total Environ.* **756**, 143732. 10.1016/j.scitotenv.2020.143732 (2021). 33279193 10.1016/j.scitotenv.2020.143732

[31] Anand, J. & Sailor, D. J. Role of pavement radiative and thermal properties in reducing excess heat in cities. *Sol. Energy* **242**, 413–423 (2022).

[32] Donthu, E. V. S. K. K., Shashwat, S., Zingre, K. T. & Wan, M. P. Development of a simplified cool coating thermal model for predicting street canyon air temperature. *Build. Environ.* **251**(May 2023), 111207. 10.1016/j.buildenv.2024.111207 (2024).

[33] Salvati, A. et al. Impact of reflective materials on urban canyon albedo, outdoor and indoor microclimates. *Build. Environ.* **207**, 108459. 10.1016/j.buildenv.2021.108459 (2022).

[34] Steemers, K., Baker, N., Crowther, D., Dubiel, J. & Nikolopoulou, M. Radiation absorption and urban texture. *Build. Res. Inf.* **26**(2), 103–112. 10.1080/096132198370029 (1998).

[35] Pawlak, W., & Fortuniak, K. Application of physical model to study effective albedo of the urban canyon. In *Proc. Fifth Int. Conf. Urban Clim. 1 - 5 Sept.*, no. c, pp. 275–278, (2003).

[36] Karimi, A. et al. Surface urban heat island assessment of a cold desert city: A case study over the Isfahan metropolitan area of Iran. *Atmosphere* **12**(10), 1368. 10.3390/atmos12101368 (2021).

[37] Aida, M. Urban albedo as a function of the urban structure—A model experiment: Part I. *Bound. Layer Meteorol.* **23**, 405–413 (1982).

[38] Alinasab, N., Mohammadzadeh, N., Karimi, A., Mohammadzadeh, R. & Gál, T. A measurement-based framework integrating machine learning and morphological dynamics for outdoor thermal regulation. *Int. J. Biometeorol.*10.1007/s00484-025-02921-8 (2025). 40259020 10.1007/s00484-025-02921-8 PMC12179017

[39] Kyriakodis, G. & Santamouris, M. Using reflective pavements to mitigate urban heat island in warm climates—Results from a large scale urban mitigation project. *Urban Clim.*10.1016/j.uclim.2017.02.002 (2017).

[40] Dimoudi, A. et al. Use of cool materials and other bioclimatic interventions in outdoor places in order to mitigate the urban heat island in a medium size city in Greece. *Sustain. Cities Soc.* **13**, 89–96. 10.1016/j.scs.2014.04.003 (2014).

[41] Al-hafiz, B., Musy, M. & Hasan, T. A study on the impact of changes in the materials reflection coefficient for achieving sustainable urban design. *Proc. Environ. Sci.* **38**, 562–570. 10.1016/j.proenv.2017.03.126 (2017).

[42] Taleghani, M. The impact of increasing urban surface albedo on outdoor summer thermal comfort within a university campus. *Urban Clim.* **24**, 175–184 (2018).

[43] Erell, E. Is urban heat island mitigation necessarily a worthy objective?. In *Proc. 33rd PLEA Int. Conf. Des. to Thrive, PLEA 2017*, vol. 2, no. November, pp. 1693–1700 (2017).

[44] Gaitani, N. et al. Improving the microclimate in urban areas: A case study in the centre of Athens. *Build. Serv. Eng. Res. Technol.* **32**(1), 53–71 (2011).

[45] Santamouris, M. et al. Using cool paving materials to improve microclimate of urban areas e Design realization and results of the fl isvos project. *Build. Environ.* **53**, 128–136. 10.1016/j.buildenv.2012.01.022 (2012).

[46] Fairuz, M., Jones, P. J., Gwilliam, J. & Salleh, E. An evaluation of outdoor and building environment cooling achieved through combination modi fi cation of trees with ground materials. *Build. Environ.* **58**, 245–257. 10.1016/j.buildenv.2012.07.012 (2012).

[47] Fang Kevin, W. K., Jon, C., & Jeremy, S. Reductions in ground level ozone pollution through urban heat 1. Island mitigation strategies including rehabbing land occupied for transportation related uses: case study of Fresno, CA. in *90th annual meeting of the Transportation Research Board. 6 Transportation and Air Quality (ADC20).* (2011).

[48] Takahashi, T. Challenge for cool city Tokyo, Osaka, pp. 12–13, (2011).

[49] Slag cement smoothes out Detroit metro airport terminal expansion. http://www.slagcement.org/News/Story_DetroitMetroAirport.html.

[50] IEC: green parking lot case study, *Heifer International Inc.*, 2007. http://www.epa.gov/region6/6sf/pdffiles/heiferparkingstudy.pdf.

[51] EPA design/construction of a permeable pavement demonstration site at the Edison Environmental Center. www.epa.gov.

[52] Taha, H. Meso-urban meteorological and photochemical modeling of heat island mitigation. *Atmos. Environ.* **42**(38), 8795–8809. 10.1016/j.atmosenv.2008.06.036 (2008).

[53] Zhou, Y., & Shepherd, Æ. J. M. Conditions and potential mitigation strategies. pp. 639–668. 10.1007/s11069-009-9406-z (2010).

[54] Rosenfeld, A. H., Akbari, H., Romm, J. J. & Pomerantz, M. Cool communities: Strategies for heat island mitigation and smog reduction. *Energy Build.* **28**(1), 51–62 (1998).

[55] Millstein, D. & Menon, S. Regional climate consequences of large-scale cool roof and photovoltaic array deployment. *Environ. Res. Lett.* **6**(3), 034001. 10.1088/1748-9326/6/3/034001 (2011).

[56] Taha, H. Urban surface modification as a potential ozone air-quality improvement strategy in California: A mesoscale modelling study. *Bound. Layer Meteorol.* **127**(2), 219–239. 10.1007/s10546-007-9259-5 (2008).

[57] Guntor, A. et al. Thermal performance of developed coating material as cool pavement material for tropical regions. *J. Mater. Civ. Eng.* **26**(4), 755–760. 10.1061/(ASCE)MT.1943-5533.0000859 (2014).

[58] Work, R. Lawrence Berkeley National Laboratory. (1992).

[59] Berkeley, L. Lawrence Berkeley National Laboratory (2003).

[60] Lawrence, E. O., Pomerantz, M., Ppn, B., & Akbari, H. The effect of pavements ‘temperatures on air temperatures in large cities ellvironmentfll energy (2000).

[61] Kinouchi K. M., Yoshinaka, T., Fukae, N., Development of cool pavement with dark colared high albedo coating. In *Proc. 5th Conf. urban Environment* (2004).

[62] Wan, W. C., Hien, W. N., Ping, T. P. & Aloysius, A. Z. W. A study on the effectiveness of heat mitigating pavement coatings in Singapore. *J. Heat Island Inst. Int.* **7**(2), 238–247 (2012).

[63] Carnielo, E. & Zinzi, M. Optical and thermal characterisation of cool asphalts to mitigate urban temperatures and building cooling demand. *Build. Environ.* **60**, 56–65. 10.1016/j.buildenv.2012.11.004 (2013).

[64] Synnefa, A., Santamouris, M. & Livada, I. J. S. E. A study of the thermal performance of reflective coatings for the urban environment. *Sol. Energy* **80**(8), 968–981. 10.1016/j.solener.2005.08.005 (2006).

[65] Isabirye, M. et al. How do social values and norms affect architecture of the Turkish house?. *INTECH* **171**, 13 (2012).

[66] Yasushi, K., Takeshi, K. & Hiroshi, O. Field measurements and heat budget analysis reduction of sensible heat emission by cool pavement. *J. Environ. Eng.* **73**(628), 791–797 (2008).

[67] Nishioka, M., Nabeshima, M., Wakama, S. & Ueda, J. Effects of surface temperature reduction and thermal environment on high albedo coating asphalt pavement. *J. Heat Island Inst. Int.* **1**, 46–52 (2006).

[68] Belkovitz, J. Can nanotechnology in concrete improve our roadways. *Emerald Cities Sustain. Conf.*, (2011).

[69] de B. A. van Bijsterveld WT, “Structural aspects of pavement heating and cooling systems.,” in *3rd International symposium on finite elements*, 2002.

[70] K. K. Kawakami Atsushi, “Development of a cool pavement for mitigating the urban heat island effect in Japan.,” in *1st International symposium on asphalt pavements and environment.*, Zurich, Switzerland: International Society for Asphalt Pavements, 2008.

[71] Karlessi, T., Santamouris, M., Apostolakis, K., Synnefa, A. & Livada, I. Development and testing of thermochromic coatings for buildings and urban structures. *Sol. Energy* **83**(4), 538–551. 10.1016/j.solener.2008.10.005 (2009).

[72] Cao, X. et al. Cooling principle analyses and performance evaluation of heat-reflective coating for asphalt pavement. *J. Mater. Civ. Eng.*10.1061/(ASCE)MT.1943-5533.0000256 (2011).

[73] Sha, A., Liu, Z., Tang, K. & Li, P. Solar heating reflective coating layer (SHRCL) to cool the asphalt pavement surface. *Constr. Build. Mater.* **139**, 355–364. 10.1016/j.conbuildmat.2017.02.087 (2017).

[74] Deevi, B. & Chundeli, F. A. Quantitative outdoor thermal comfort assessment of street: A case in a warm and humid climate of India. *Urban Clim.* **34**, 100718. 10.1016/j.uclim.2020.100718 (2020).

[75] Nasrollahi, N., Hatami, M., Khastar, S. R. & Taleghani, M. Numerical evaluation of thermal comfort in traditional courtyards to develop new microclimate design in a hot and dry climate. *Sustain. Cities Soc.* **35**, 449–467. 10.1016/j.scs.2017.08.017 (2017).

[76] Apreda, C., Reder, A. & Mercogliano, P. Urban morphology parameterization for assessing the effects of housing blocks layouts on air temperature in the Euro-Mediterranean context. *Energy Build.* **223**, 110171. 10.1016/j.enbuild.2020.110171 (2020).

[77] Teshnehdel, S., Mirnezami, S., Saber, A., Pourzangbar, A. & Olabi, A. G. Data-driven and numerical approaches to predict thermal comfort in traditional courtyards. *Sustain. Energy Technol. Assessments* **37**(November), 100569. 10.1016/j.seta.2019.100569 (2020).

[78] Schwarz, N., Lautenbach, S. & Seppelt, R. Exploring indicators for quantifying surface urban heat islands of European cities with MODIS land surface temperatures. *Remote Sens. Environ.* **115**(12), 3175–3186. 10.1016/j.rse.2011.07.003 (2011).

[79] Taha, H. Urban climates and heat islands: Albedo, evapotranspiration, and anthropogenic heat. *Energy Build.* **25**(2), 99–103. 10.1016/s0378-7788(96)00999-1 (1997).

[80] Rizwan, A. M. & Dennis, L. Y. A review on the generation, determination and mitigation of Urban Heat Island. *J. Environ. Sci.* **20**(1), 120–128. 10.1016/S1001-0742(08)60019-4 (2008). 10.1016/s1001-0742(08)60019-4 18572534

[81] Perini, K., Chokhachian, A., Dong, S. & Auer, T. Modeling and simulating urban outdoor comfort: Coupling ENVI-Met and TRNSYS by grasshopper. *Energy Build.* **152**, 373–384. 10.1016/j.enbuild.2017.07.061 (2017).

[82] Błażejczyk, K., Kuchcik, M., Błażejczyk, A., Milewski, P. & Szmyd, J. Assessment of urban thermal stress by UTCI–experimental and modelling studies: An example from Poland. *DIE ERDE J. Geogr. Soc. Berlin* **145**(1–2), 16–33. 10.12854/erde-145-3 (2014).

[83] Schrijvers, P. J. C., Jonker, H. J. J., De Roode, S. R. & Kenjereš, S. The effect of using a high-albedo material on the Universal Temperature Climate Index within a street canyon. *Urban Clim.* **17**, 284–303. 10.1016/j.uclim.2016.02.005 (2016).

[84] Shooshtarian, S., Lam, C. K. C. & Kenawy, I. Outdoor thermal comfort assessment: A review on thermal comfort research in Australia. *Build. Environ.* **177**, 106917. 10.1016/j.buildenv.2020.106917 (2020).

[85] Honjo, T. Thermal comfort in outdoor environment. *Global Environ. Res.* **13**(2009), 43–47 (2009).

[86] Li, H., He, Y. & Harvey, J. Human thermal comfort: Modeling the impact of different cool pavement strategies. *Transp. Res. Rec.* **2575**, 92–102. 10.3141/2575-10 (2016).

[87] Huang, X., Song, J., Wang, C. & Chan, P. W. Realistic representation of city street-level human thermal stress via a new urban climate-human coupling system. *Renew. Sustain. Energy Rev.* **169**, 112919 (2022).

[88] Lai, D., Liu, W., Gan, T., Liu, K. & Chen, Q. A review of mitigating strategies to improve the thermal environment and thermal comfort in urban outdoor spaces. *Sci. Total Environ.* **661**, 337–353. 10.1016/j.scitotenv.2019.01.062 (2019). 30677681 10.1016/j.scitotenv.2019.01.062

[89] Abdallah, A. S. H. & Mahmoud, R. M. A. Urban morphology as an adaptation strategy to improve outdoor thermal comfort in urban residential community of new assiut city, Egypt. *Sustain. Cities Soc.* **78**, 103648. 10.1016/j.scs.2021.103648 (2022).

[90] Chen, L., Zhang, Y., Han, J. & Li, X. An investigation of the influence of ground surface properties and shading on outdoor thermal comfort in a high-altitude residential area. *Front. Archit. Res.* **10**(2), 432–446. 10.1016/j.foar.2020.12.005 (2021).

[91] Xu, M., Hong, B., Jiang, R., An, L. & Zhang, T. Outdoor thermal comfort of shaded spaces in an urban park in the cold region of China. *Build. Environ.* **155**(March), 408–420. 10.1016/j.buildenv.2019.03.049 (2019).

[92] Farhadi, H., Faizi, M. & Sanaieian, H. Mitigating the urban heat island in a residential area in Tehran: Investigating the role of vegetation, materials, and orientation of buildings. *Sustain. Cities Soc.* **46**, 101448. 10.1016/j.scs.2019.101448 (2019).

[93] Mohammad, P. et al. Evaluating the role of the albedo of material and vegetation scenarios along the urban street canyon for improving pedestrian thermal comfort outdoors. *Urban Clim.* **40**, 100993. 10.1016/j.uclim.2021.100993 (2021).

[94] Karimi, A. et al. New developments and future challenges in reducing and controlling heat island effect in urban areas. *Environ. Dev. Sustain.* **25**(10), 10485–10531. 10.1007/s10668-022-02530-0 (2023).

[95] Evola, G., Naboni, E., Margani, G., Magri’, C. modeling outdoor thermal comfort in urban canyons: Presentation and validation of a novel comprehensive workflow. In *Proceedings of Building Simulation 2019: 16th Conference of IBPSA*, IBPSA, pp. 3288–3295. 10.26868/25222708.2019.210402 (2020)

[96] Kamel, T. M. A new comprehensive workflow for modelling outdoor thermal comfort in Egypt. *Sol. Energy* **225**(April), 162–172. 10.1016/j.solener.2021.07.029 (2021).

[97] Climate One Building. https://www.climate.onebuilding.org/ (accessed Jul. 29, 2024).

[98] Africa-Region 1, Climate One Building. Retrieved from 29 July 2024. https://climate.onebuilding.org/WMO_Region_1_Africa/EGY_Egypt/QH_Al_Qahirah_Cairo/EGY_QH_Cairo.Intl.AP.623660_TMYx.zip.

[99] Reuy-Lung, H., Chen, C.-P., Feng-Yi, L., Wen-Mei, S., & Kuo-Tsang, H. Applicability of ASHRAE Standard 55 and EN 15251 adaptive thermal comfort models in hot-and-humid climate. In *Environment and Health—Bridging South, North, East and West: Conference of ISEE, ISES and ISIAQ*, 2013. [Online]. Available: https://ehp.niehs.nih.gov/doi/10.1289/isee.2013.P-2-12-01

[100] Abd Elraouf, R., Elmokadem, A., Megahed, N., Eleinen, O. A. & Eltarabily, S. The impact of urban geometry on outdoor thermal comfort in a hot-humid climate. *Build. Environ.* **225**, 109632. 10.1016/j.buildenv.2022.109632 (2022).

[101] Aboelata, A. Reducing outdoor air temperature, improving thermal comfort, and saving buildings’ cooling energy demand in arid cities–Cool paving utilization. *Sustain. Cities Soc.* **68**, 102762. 10.1016/j.scs.2021.102762 (2021).

[102] Detommaso, M., Costanzo, V. & Nocera, F. Application of weather data morphing for calibration of urban ENVI-met microclimate models, Results and critical issues. *Urban Clim.* **38**, 100895. 10.1016/j.uclim.2021.100895 (2021).

[103] Schneider, F. A., Ortiz, J. C., Vanos, J. K., Sailor, D. J. & Middel, A. Evidence-based guidance on reflective pavement for urban heat mitigation in Arizona. *Nat. Commun.* **14**(1), 1467. 10.1038/s41467-023-36972-5 (2023). 36928319 10.1038/s41467-023-36972-5 PMC10020537

[104] Elwy, I., Ibrahim, Y., Fahmy, M. & Mahdy, M. Outdoor microclimatic validation for hybrid simulation workflow in Outdoor microclimatic validation for hybrid simulation workflow in hot arid. The climates against and field measurements hot arid climates against ENVI-met and field measurements Assessing. *Energy Proc.* **153**, 29–34. 10.1016/j.egypro.2018.10.009 (2018).

[105] Testo, Globe probe Ø 150mm, TC Type K for measuring radiant heat (0602 0743). https://www.testo.com/en/globe-probe-o-150mm-tc-type-k-for-measuring-radiant-heat/p/0602-0743.

[106] Galal, O. M., Mahmoud, H. & Sailor, D. Impact of evolving building morphology on microclimate in a hot arid climate. *Sustain. Cities Soc.* **54**, 102011. 10.1016/j.scs.2019.102011 (2020).

[107] Ma, X., Zhao, J., Zhang, L., Wang, M., & Cheng, Z. The deviation between the field measurement and ENVI-met outputs in winter—A cases study in a traditional dwelling settlement of China (2020).

[108] ISO, “ISO 14562:2005 – Laboratory Glassware – Porosity – Method of Test,” Geneva, 2005. [Online]. Available: https://www.iso.org/standard/14562.html.

[109] Extech Instruments, “Extech EasyView Indoor Air Quality Meter/Data Logger.” https://www.flir.com/products/ea80/?segment=solutions&vertical=condition+monitoring.

[110] Elprocus, “What is a Hot-Wire Anemometer & Its Working?” https://www.elprocus.com/what-is-a-hot-wire-anemometer-its-working/.

[111] Lu, Y., Rahman, M. A., Moore, N. W. & Golrokh, A. J. Lab-controlled experimental evaluation of heat-reflective coatings by increasing surface albedo for cool pavements in urban areas. *Coatings* **12**(1), 7. 10.3390/coatings12010007 (2021).

[112] Lu, Y., Qin, Y., Huang, C. & Pang, X. Albedo of pervious concrete and its implications for mitigating urban heat island. *Sustainability* **15**(10), 8222. 10.3390/su15108222 (2023).

[113] Sanjuán, M. Á., Morales, Á. & Zaragoza, A. Precast concrete pavements of high albedo to achieve the net “zero-emissions” commitments. *Appl. Sci.* **12**(4), 1955. 10.3390/app12041955 (2022).

[114] Dumais, S. & Doré, G. An albedo based model for the calculation of pavement surface temperatures in permafrost regions. *Cold Reg. Sci. Technol.* **123**, 44–52. 10.1016/j.coldregions.2015.11.013 (2016).

[115] Chen, J., Wang, H. & Zhu, H. Analytical approach for evaluating temperature field of thermal modified asphalt pavement and urban heat island effect. *Appl. Therm. Eng.* **113**, 739–748. 10.1016/j.applthermaleng.2016.11.080 (2017).

[116] Yang, J., Wang, Z. H., Kaloush, K. E. & Dylla, H. Effect of pavement thermal properties on mitigating urban heat islands: A multi-scale modeling case study in Phoenix. *Build. Environ.* **108**, 110–121. 10.1016/j.buildenv.2016.08.021 (2016).

[117] Ibrahim, Y., Kershaw, T., & Shepherd, P. Improvement of the Ladybug-tools microclimate workflow: A verification study Department of Architecture & Civil Engineering , University of Bath, Bath , UK Abstract.

[118] Qin, Y. A review on the development of cool pavements to mitigate urban heat island effect. *Renew. Sustain. Energy Rev.* **52**, 445–459. 10.1016/j.rser.2015.07.177 (2015).

[119] Fahmy, M., Ibrahim, Y., Hanafi, E. & Barakat, M. Would LEED-UHI greenery and high albedo strategies mitigate climate change at neighborhood scale in Cairo, Egypt?. *Build. Simul.* **11**, 1273–1288 (2018).

[120] Ibrahim, Y., Kershaw, T., Shepherd, P. & Coley, D. On the optimisation of urban form design, energy consumption and outdoor thermal comfort using a parametric workflow in a hot arid zone. *Energies* **14**(13), 4026 (2021).

[121] Rossi, F. et al. Experimental evaluation of urban heat island mitigation potential of retro-reflective pavement in urban canyons. *Energy Build.* **126**, 340–352. 10.1016/j.enbuild.2016.05.036 (2016).

[122] Chiatti, C., Fabiani, C., Huang, X., Bou-zeid, E. & Laura, A. Exploring the potential of phosphorescence for mitigating urban overheating: First time representation in an Urban Canopy Model. *Appl. Energy* **362**(November 2023), 122984 (2024).

[123] Kousis, I., Fabiani, C. & Pisello, A. L. A study on the thermo-optical behaviour of phosphorescent coatings for passive cooling applications. *E3S Web Conf.* **06002**, 06002 (2021).

[124] Huang, X., Bou-Zeid, E., Pigliautile, I., Pisello, A. L. & Mandal, J. Optimizing retro-reflective surfaces to untrap radiation and cool cities. *Nat. Cities* **1**(4), 275–285. 10.1038/s44284-024-00047-3 (2024).

[125] Raman, A. P., Anoma, M. A., Zhu, L., Rephaeli, E. & Fan, S. Passive radiative cooling below ambient air temperature under direct sunlight. *Nature*10.1038/nature13883 (2014). 25428501 10.1038/nature13883

